# Research progress of small molecule targeted drugs for non-small cell lung cancer

**DOI:** 10.3389/fonc.2026.1847493

**Published:** 2026-07-15

**Authors:** Tingxiang Qiu, Xin Huang

**Affiliations:** 1 College of Health Management, Shanghai Jian Qiao University, Shanghai, China; 2 School of Medical College, Xi’an Peihua University, Xi’an, China

**Keywords:** drug resistance, non-small cell lung cancer, small molecule, targeted drug, targeted therapy

## Abstract

Non-small cell lung cancer (NSCLC) constitutes approximately 85% of all lung cancer cases and remains a major clinical challenge with a poor overall prognosis. Molecular targeted therapy has thus emerged as a cornerstone of its treatment. This review summarizes the research progress of advances in small-molecule targeted agents against major driver oncogenes in NSCLC, including epidermal growth factor receptor (EGFR), anaplastic lymphoma kinase (ALK), and Kirsten rat sarcoma viral oncogene homolog (KRAS). We elaborate on the mechanism of action, clinical efficacy and treatment-related adverse events (TRAEs) of each generation of inhibitors, while updating the developmental status of innovative therapies and emerging. Notably, this article further integrates clinical data of investigational small-molecule targeted drugs that address emerging oncogenic drivers such as rat sarcoma viral oncogene homolog (RAS), human epidermal growth factor receptor 2 (HER2), and v-src avian sarcoma viral oncogene homolog (SRC), which have demonstrated promising clinical activity in early-phase trials. These findings highlight potential avenues for future development of NSCLC-targeted therapies. Despite iterative optimization across multiple generations, current targeted drugs for NSCLC still face prominent limitations, including acquired drug resistance and insufficient penetration across the blood-brain barrier (BBB). Moving forward, future research should prioritize accelerating the clinical translation of investigational drugs, exploring combination treatment regimens, identifying novel molecular targets, and optimizing the overall system of molecular targeted therapy. These efforts will help further improve clinical outcomes for patients with NSCLC.

## 1 Introduction

Primary bronchogenic carcinoma, clinically termed lung cancer, is a malignant neoplasm derived from the bronchial epithelium or glands. Ranking among the most prevalent human malignancies, lung cancer remains the leading cause of cancer-related mortality globally (1). Non-small cell lung cancer (NSCLC) constitutes 85% of total lung cancer cases, with predominant pathological subtypes including adenocarcinoma (40%), squamous cell carcinoma (30%), large cell undifferentiated carcinoma (15%), and a small proportion of rare pathological variants (2). Characterized by prominent molecular features, NSCLC exhibits strong invasive and metastatic propensity, as well as inherent chemoresistance. Such aggressive biological traits pose substantial challenges to clinical treatment, resulting in a dismal prognosis with a 5-year overall survival rate of merely 15% (3, 4). Currently, surgery-centered multiple therapies serve as the standard treatment for lung cancer. Nevertheless, nearly 80% patients present with advanced diseases at diagnosis, precluding curative resection. Platinum-doublet chemotherapy has long been the conventional first-line therapeutic regimen for patients with advanced NSCLC. Although this regimen improves 1-year survival from 20% to 29%, its objective response rate hovers at only 30%-40%. Furthermore, chemotherapy is associated with severe systemic toxicities, which are poorly tolerated by the majority of patients. This frequently results in premature treatment cessation, which restricts the widespread clinical use of this conventional therapy (5). Mounting clinical evidence has demonstrated the unique advantages of molecular targeted therapy, including potent anti-tumor activity and a favorable safety profile. In NSCLC, targeted agents exert high selectivity toward malignant lesions, reducing damage to normal tissues, lowering the incidence of drug-related TRAEs, and enhancing clinical tolerability. The progressive clinical integration of diverse targeted agents has markedly improved the prognosis and quality of life of advanced NSCLC patients, underscoring its substantial clinical value (6). In recent years, extensive clinical investigations have been conducted on oncogenic drivers in NSCLC, such as epidermal growth factor receptor (*EGFR*), anaplastic lymphoma kinase (*ALK*), Kirsten rat sarcoma viral oncogene homolog (*KRAS*), c-ros oncogene 1 receptor tyrosine kinase (*ROS1*), V-Raf murine sarcoma viral oncogene homolog B (*BRAF*), rearranged during transfection (*RET*), and mesenchymal–epithelial transition factor (*MET*). These studies have yielded substantial progress, providing critical support for the clinical implementation and refinement of targeted therapy (4). Additionally, emerging oncogenic drivers, including rat sarcoma viral oncogene homolog (*RAS*), human epidermal growth factor receptor 2 (*HER2*), and rous sarcoma oncogene (*SRC*), have also attracted growing interest in recent years. Investigations into targeted agents against these emerging drivers are underway, holding promise for novel breakthroughs in NSCLC treatment. This paper summarizes the major small molecule targeted agents for NSCLC and outlines future directions in this field, aiming to provide a reference for clinical practice and drug development in NSCLC.

## 2 Small molecule targeted drugs targeting EGFR

Among driver oncogenes, *EGFR*, also known as erythroblastic leukemia viral oncogene homolog 1 (*ErbB1*)/human epidermal growth factor receptor 1 (*HER1*), is a receptor tyrosine kinase (RTK) belonging to the ErbB family of tyrosine kinase receptors (7). It comprises three structural domains: an extracellular ligand-binding region, a transmembrane segment (8). When ligands such as epidermal growth factor bind to the extracellular domain of EGFR, signals are transmitted through the transmembrane segment to the intracellular kinase domain, activating the tyrosine kinase domain and triggering downstream signaling pathways including the RAS/rapidly accelerated fibrosarcoma (RAF)/mitogen−activated protein kinase (MAPK), and phosphoinositide 3-kinase (PI3K)-protein kinase B (AKT) signaling axes. These pathways orchestrate key physiological processes such as cell proliferation, growth, anti-apoptosis and migration. Abnormal overexpression of EGFR is closely associated with tumor invasion, metastasis and poor prognosis (9). Contemporary preclinical and clinical evidence confirms that EGFR drives malignant tumor initiation and progression by modulating cell proliferation, apoptosis and promoting angiogenesis (10). Inhibiting EGFR tyrosine kinase activity thus effectively halts malignant progression, establishing EGFR as a pivotal therapeutic target for NSCLC. In recent years, research, developments and clinical applications of EGFR tyrosine kinase inhibitors (TKIs) have developed rapidly. At present, the first-, second-, and third-generations of EGFR-TKIs have gained regulatory approval in China, while the fourth-generation of EGFR-TKIs has gradually progressed to clinical trials (11). Small-molecule targeted agents targeting EGFR are summarized in **Table 1**.

**Table 1:** Summary of small-molecule targeted drugs for EGFR.

Class	Drug	Target	Adaptation disease	Adverse reaction (Grade ≥ 3)	Central nervous system penetration capacity
First generation	Gefitinib	EGFR	First-line treatment for metastatic NSCLC harboring EGFR exon 19 deletion/exon 21 L858R mutations (12).	Grade 3 or higher diarrhea, severe rash, severe keratitis: suspend medicine within 14 days, resume after symptoms improve to grade 1. Confirmed interstitial lung disease (ILD), gastrointestinal (GI) perforation, refractory severe keratitis, severe liver injury: discontinue medicine permanently (12).	Inherent limitation of limited blood-brain barrier penetration (13).
	Erlotinib	EGFR	Metastatic NCLSC with specified EGFR mutations; In combination with gemcitabine for advanced and metastatic pancreatic cancer (14).	Grade 3 or above diarrhea, vomiting, rash, keratitis, liver and kidney injury, severe fatigue and severe infection: suspend treatment for no more than 14 days, resume after improvement to grade 1. Confirmed ILD, respiratory failure, G perforation, severe exfoliative dermatitis, refractory severe keratitis and severe thrombosis: discontinue treatment permanently (14).	Having a reliable ability to penetrate the central nervous system is an important advantage for its use in combination with whole-brain radiotherapy for brain metastases of non-small cell lung cancer (15).
	Icotinib	EGFR	Monotherapy: EGFR-sensitizing NSCLC, post platinum chemo failure, adjuvant for stage II-IIIA post-resection; avoid EGFR wild-type patients (16).	Chiefly severe rash, diarrhea, elevated liver enzymes; occasional severe paronychia, stomatitis, cytopenia, ILD, renal injury and fatigue. Pause and treat symptoms for Grade ≥ 3; stop permanently for Grade 4 (16).	The blood-brain barrier penetration effect is good, and it can effectively act on the intracranial metastatic lesions of lung cancer (17).
Second generation	Afatinib	EGFR	Untreated EGFR-mutant metastatic non-squamous NSCLC; metastatic squamous NSCLC post platinum chemotherapy failure (18)	GI and skin reactions, hypokalemia, lymphopenia, elevated liver enzymes. Severe risks: ILD, pneumonia, acute renal failure and death; rare severe pancreatitis. These GI and skin problems commonly lead to dose interruption or discontinuation (18).	The blood-brain barrier has poor permeability, making it difficult to achieve an effective drug concentration within the brain (19).
	Dacomitinib	EGFR	First-line treatment of metastatic NSCLC with EGFR exon 19 deletion or L858R mutation (20).	Frequent severe diarrhea, rash, paronychia and stomatitis. Fatal ILD may occur; permanently discontinue once confirmed (20).	The blood-brain barrier penetration ability is excellent, the intracranial anti-tumor effect is powerful and the dose tolerance is good. It is one of the preferred drugs for treating brain metastases of EGFR-mutated lung cancer (21).
Third generation	Osimertinib	EGFR	Adjuvant for EGFR-mutant resected NSCLC; monotherapy for stable unresectable stage III NSCLC; first-line alone or with pemetrexed/platinum; later-line for T790M-positive post-TKI failure (22).	Pneumonia, QT interval prolongation, GI and skin damage, blood count drop and electrolyte imbalance. Chemotherapy combination increases severe myelosuppression. ILD causes permanent withdrawal; rare severe issues: Stevens-Johnson Syndrome, Toxic Epidermal Necrolysis, aplastic anemia, cardiomyopathy (22).	Structural modification effectively overcomes the obstruction of excretory proteins, with a remarkable increase in the level of drug exposure within the brain, and a significant advantage in central penetration (19).
	Lazertinib	EGFR	Lazertinib + amivantamab: first-line for U.S. Food and Drug Administration (FDA)-confirmed EGFR 19del/L858R advanced NSCLC (23).	Rash, nail toxicity, venous thromboembolism; plus infusion reaction, fatigue, edema, diarrhea, stomatitis. Lab anomalies: hypoalbuminemia, hyponatremia, elevated liver enzymes, hypokalemia, anemia. Severe toxicities (may be fatal): venous thromboembolism, pneumonia, rash, ILD (23).	The blood-brain barrier has excellent permeability, enabling the stable formation of effective drug concentrations within the brain, making it suitable for patients with lung cancer brain/meningeal metastases who have failed first and second-generation EGFR-targeted therapies (24).

### 2.1 First-generation EGFR-TKIs

First-generation EGFR-TKIs comprise reversible small-molecule quinazoline compounds, mainly including Gefitinib (trade name: Iressa; hereafter referred to as Gefitinib), Erlotinib (trade name: Tarceva; hereafter referred to as Erlotinib), and Icotinib (trade name: Conmana; hereafter referred to as Icotinib), a domestically developed agent with independent intellectual property rights. These drugs competitively bind the adenosine triphosphate (ATP) binding site of EGFR kinase domain, blocking dysregulated downstream signaling pathways and thereby exerting an anti-tumor activity (11). These can significantly improve the objective response rate and survival time of newly treated EGFR mutation-positive NSCLC patients (7).

Gefitinib was the first EGFR TKI approved for NSCLC treatment. It binds the EGFR tyrosine kinase domain competitively, inhibiting the EGFR signaling pathway to suppress tumor cell proliferation, induce apoptosis, and reduce tumor burdens (25). Although over half of *EGFR*-mutant NSCLC patients respond effectively to initial Gefitinib therapy, most eventually develop acquired drug resistance, mainly due to the occurrence of T790M mutation, which results in a median progression-free survival (mPFS) of merely 12–14 months in patients. Approximately 30% of patients are ineligible for subsequent lines of therapy due to rapid tumor progression (26). To overcome drug resistance and improve outcomes, clinical studies have gradually explored Gefitinib-based combination strategies, aiming to break the efficacy ceiling of monotherapy. Metformin, a widely used anti-diabetic agent, has attracted increasing clinical interest for its anti-tumor properties in recent years. A large number of clinical studies show anti-tumor effects by inhibiting gluconeogenesis, reducing inflammatory responses and modulating the cell cycle progression (27). A study by Chen et al. reported that the combination of metformin and Gefitinib achieved an overall response rate of 96.1% in NSCLC patients. Post-treatment serum levels of squamous cell carcinoma antigen and cytokeratin 19 fragment antigen 21–1 in patients decreased to (2.81 ± 0.33) ng/L and (5.31 ± 0.57) μg/L, respectively. These results demonstrated that the combination regimen can effectively inhibit the tumor cell growth, metastasis, angiogenesis and induce apoptosis (28). A single-center Chinese clinical trial enrolled 100 patients with advanced NSCLC admitted to Shangqiu Municipal Hospital between January 2017 and February 2019. All patients were randomized 1:1 to a control group (n = 50) receiving G protein (GP) regimen chemotherapy and the study group (n = 50) receiving GP regimen chemotherapy combined with Gefitinib. The results showed that the objective response rate (ORR) of the study group was 84.00%, which was significantly higher than 64.00% in the control group, and the disease control rate (DCR) was also significantly increased in the study group. After treatment, the levels of tumor markers, including neuron-specific enolase (NSE), carbohydrate antigen 72-4 (CA72-4) and carbohydrate antigen 19-9 (CA19-9) were significantly lower in the study group, alongside more pronounced improvements in immune function parameters[CD4^+^ T lymphocyte (CD4^+^) and cytotoxic T lymphocyte (CD8^+^)]. The study group achieved a quality of life score of 84.13 ± 4.78, which was significantly higher than the 60.15 ± 5.61 points of the control group. The total incidence of adverse reactions was 38.00% in both groups, with no statistically significant difference. This study demonstrates that Gefitinib combined with conventional chemotherapy yields superior efficacy with favorable safety in Chinese patients with advanced NSCLC, while significantly improving quality of life (29).

As an EGFR TKI, Erlotinib inhibits EGFR-mediated intracellular tyrosine kinase phosphorylation, abrogating downstream signaling to suppress tumor cell proliferation and prolong patient survival (30). A controlled trial involving 69 patients with advanced NSCLC showed no statistically significant differences in the ORR (35.29% vs 31.43%) and DCR (97.06% vs 85.71%) between the Erlotinib monotherapy and conventional chemotherapy group (*P* > 0.05). However, the former achieved a significantly longer mPFS (12.6 months vs 4.2 months; *P* < 0.001), with efficacy unaffected by clinical factors including gender or age (*P* > 0.05) (31). The above study initially confirmed the clinical value of Erlotinib monotherapy for advanced NSCLC. Chemotherapy, as the traditional core treatment for this disease, has limited efficacy in middle and advanced patients, providing an important space for the clinical application of targeted therapy. A combination of the two has become an important direction to optimize treatment for middle and advanced patients. In clinical practice, common NSCLC chemotherapy drugs including platinum-based drugs {e.g., cisplatin [cell cycle non-specific drugs that exert anti-tumor effects by inhibiting tumor cell deoxyribonucleic acid (DNA) replication]} (32) and paclitaxel (a first-line chemotherapy drug recommended in current guidelines that blocks tumor cell mitosis by stabilizing tubulin). However, advanced tumors exhibit high invasiveness, and single chemotherapy often fails to achieve the desired outcomes (33, 34). Against this background, molecular targeted therapy, with its precision targeting of driver gene alterations and effective induction of tumor cell apoptosis, has become an important strategy for middle and advanced malignant tumors, with Erlotinib as a prototypical agent (6). The multicenter phase III OPTIMAL trial verified that for first-line monotherapy in *EGFR-*mutant advanced NSCLC, Erlotinib yielded a mPFS of 13.1 (95% CI:10.58–16.53) months, which was significantly superior to the gemcitabine plus carboplatin chemotherapy(mPFS: 4.6 months; 95% CI:4.21–5.42; hazard ratio (HR) = 0.16, 95% CI: 0.10–0.26, *P*<0.0001) (35). A prospective randomized controlled study of 100 patients with stage III-IV NSCLC conducted from January 2021 to January 2023 further confirmed the clinical benefits of erlotinib combined with chemotherapy. On the basis of first-line chemotherapy with cisplatin combined with nab-paclitaxel, Erlotinib (0.15g daily) was added in this study. The results showed that the levels of serum tumor markers such as carcinoembryonic antigen (CEA) in the combined treatment group decreased significantly, with superior ORR (64.00% vs 42.00%), DCR (70.00% vs 50.00%), 1-year survival rate (74.00% vs 52.00%), mPFS (10.3 months vs 9.2 months) and median overall survival (mOS) (11.6 months vs 10.7 months) compared with chemotherapy alone group (all *P* < 0.05) (36).

Icotinib selectively inhibits the combination of ATP and EGFR-tyrosine kinase (TK), reduces the activity of EGFR-TK, and thus effectively blocks the EGFR signaling transduction (37). As an original Chinese EGFR-TKI, Icotinib has been validated in multiple phase III clinical trials conducted in China. The ICOGEN phase III trial demonstrated that Icotinib achieved an ORR of 27.6%, a DCR of 75.4%, and a median progression-free survival (PFS) of 4.6 months, exhibiting non-inferior efficacy and a better safety profile compared with Gefitinib. The EVIDENCE phase III trial, focusing on postoperative adjuvant therapy, showed a median disease-free survival (DFS) of 47.0 months and a 3-year DFS rate of 63.9%, which was significantly superior to chemotherapy. The ICTAN phase III trial confirmed that 6-month and 12-month adjuvant Icotinib treatment yielded equivalent efficacy and could significantly reduce the risk of tumor recurrence. In first-line treatment settings, Icotinib presents an ORR of 55%–60% and a DCR of approximately 90%, with definite survival benefits and favorable tolerability (38).

Pemetrexed, an antifolate drug, can destroy the normal folate-dependent metabolic process in tumor cells, inhibiting the proliferation and replication of tumor cells (39). Carboplatin, a second-generation platinum-based anti-tumor drug, binds double-stranded DNA to impair replication and repair, as well as activate related signaling pathways, induce apoptosis and inhibit cancer cell proliferation (40). To explore the clinical efficacy of pemetrexed combined with Carboplatin chemotherapy in patients with advanced NSCLC progressing after first-line EGFR-TKI treatment, and to compare the therapeutic effects of icotinib monotherapy, icotinib combined with pemetrexed plus carboplatin. A total of 84 patients with advanced NSCLC presenting slow disease progression following first−line EGFR−TKI therapy were enrolled and equally randomized 1:1:1 into three groups (n = 28 each): Group A (Icotinib monotherapy), Group B (pemetrexed plus carboplatin chemotherapy), and Group C (combination of Icotinib, pemetrexed and carboplatin). All patients received a 42−day treatment course. No statistically significant difference was observed in DCR among the three groups (71.4% for Group A, 85.7% for Group B, 78.6% for Group C, *P* > 0.05). Complete response was not achieved in any patient across all cohorts; partial response was absent in Group A, while the partial response rates reached 28.6% and 25.0% in Group B and Group C, respectively. After treatment, serum levels of CEA and cytokeratin 19 fragment (CYFRA21−1) were significantly lower in Group B (CEA: 5.5 ± 0.4 μg/L; CYFRA21−1: 2.9 ± 0.2 μg/L) and Group C (CEA: 5.1 ± 0.6 μg/L; CYFRA21−1: 2.5 ± 0.3 μg/L) compared with Group A (CEA: 7.1 ± 1.2 μg/L; CYFRA21−1: 3.6 ± 0.5 μg/L, *P* < 0.05) (41).

The primary TRAEs of Gefitinib and Erlotinib include rash, GI dysfunction and mild hepatotoxicity, with a low incidence rate and tolerable to most patients (11). TRAEs associated with icotinib in advanced NSCLC are mainly mild to moderate. The most frequent adverse event is skin rash, followed by diarrhea, pruritus and mild hepatotoxicity. Few patients discontinue treatment due to TRAEs. Overall, icotinib exhibits a well-tolerated and manageable safety profile (42).

### 2.2 Second-generation EGFR-TKIs

To address acquired resistance to first-generation EGFR-TKIs, researchers have developed second-generation irreversible EGFR-TKIs, represented by Afatinib (trade name: Gilotrif; hereafter referred to as Afatinib) and Dacomitinib (trade name: Vizimpro; hereafter referred to as Dacomitinib). These agents share structural homology with Gefitinib and Erlotinib but incorporate a novel side chain enabling covalent binding to the C797 residue of EGFR, thereby exerting irreversible inhibition of EGFR tyrosine kinase. They also concomitantly target the HER2 receptor, augmenting anti-tumor efficacy (11).

Seventy patients with locally advanced NSCLC treated at Fuxin Mining General Hospital of Liaoning Health Industry Group (China) received Afatinib combined with chemotherapy regimens. The results showed that the ORR was 11.43% and the DCR was 58.57%. After treatment, serum levels were as follows: CYFRA21−1 (4.25 ± 1.12) μg/L, CEA (93.58 ± 9.75) μg/L, carbohydrate antigen 125 (CA125) (60.24 ± 4.34) U/ml, and vascular endothelial growth factor (VEGF) (373.47 ± 48.38) ng/L. During treatment, adverse event rates were: hepatorenal impairment (21.43%), gastrointestinal adverse reactions (65.71%), fever (28.81%), anemia (45.71%), and skin rash (22.86%). Most adverse events were grade I–II, indicating an acceptable overall safety profile (43). The pivotal international multicenter Phase III ARCHER 1050 trial of Dacomitinib enrolled 231 Chinese patients, representing over 50% of the global cohort of 452 patients. In the Chinese subgroup, the mPFS reached 18.4 months, versus a global mPFS of 14.7 months and mOS of 34.1 months; the Asian population achieved a mPFS of 16.5 months and mOS of 37.7 months. Dacomitinib demonstrated statistically superior efficacy versus Gefitinib. Diarrhea and skin rash were the common adverse events, which were manageable with dose adjustments (44).

Several clinical studies have conducted head-to-head comparisons of first- and second-generation EGFR-TKIs. The Phase IIB trial compared the efficacy of Afatinib and Gefitinib in treatment-naive advanced NSCLC patients with common *EGFR* mutations (e.g., exon 19 deletion, L858R). The results showed that Afatinib significantly improved the PFS and ORR of patients, but there was no significant statistical difference in overall survival (OS) between the two groups (45). The ARCHER-1050 randomized open-label phase III trial further confirmed that Dacomitinib treatment yielded significantly better PFS and OS than Gefitinib in terms of newly diagnosed *EGFR* mutation-positive advanced NSCLC patients (46, 47).

Notably, compared with the first-generation agents, second-generation EGFR-TKIs are associated with a higher incidence of TRAEs, mainly manifested as diarrhea, rash, etc. Some patients require dose adjustments to maintain tolerability, which limits their clinical utility to a certain extent (7).

### 2.3 Third-generation EGFR-TKIs

While first- and second-generation EGFR-TKIs exhibit significant efficacy in the initial treatment of advanced NSCLC, most patients develop acquired resistance after continuous medication for 6–13 months. The *EGFR* exon 20 T790M gene mutation accounts for 40% - 55% of resistance cases (48). The gatekeeper T790M mutation increases the affinity of the EGFR kinase domain for ATP, which impairs the binding capacity of first- and second-generation EGFR-TKIs to their target and ultimately leads to therapeutic failure. Several clinical studies have evaluated the combination of afatinib and cetuximab for patients with T790M-mediated acquired resistance; however, severe treatment-related adverse events (TRAEs) and dose-limiting toxicities hinder its clinical application, making it unable to fundamentally overcome T790M resistance. To address this unmet clinical need, third-generation irreversible EGFR-TKIs with high selectivity against mutant EGFR have been developed. Developed specifically for patients harboring the T790M resistance mutation, these agents covalently and irreversibly bind EGFR carrying sensitizing alterations (exon 19 deletions, L858R) as well as the T790M mutation, while exerting minimal inhibitory effects on wild-type EGFR. Moreover, their active metabolites generated after *in vivo* metabolism also exhibit potent anti-tumor activity (49).

Osimertinib (trade name: Tagrisso; hereafter referred to as Osimertinib), a third-generation EGFR-TKI developed by AstraZeneca, is currently the most widely used third-generation EGFR-TKI clinically. A randomized double-blind phase III clinical trial conducted by Duma et al. confirmed that Osimertinib achieved a mPFS of 38.6 months in advanced NSCLC, significantly exceeding the 31.8 months in the Gefitinib group, with no obvious differences in drug safety between the two groups (50). The results of the FLAURA study further showed that Osimertinib prolonged PFS versus first-generation EGFR-TKIs(18.9 months vs 10.2 months), while effectively reducing the risk of disease progression (51). A single-center retrospective study in China enrolled 74 *EGFR*-mutant NSCLC patients receiving first-line Osimertinib treatment. The mPFS among Chinese patients was 16.3 months. Patients with stage IIIA/IVA disease achieved a median PFS of 25.1 months, which was significantly longer than 15.0 months in patients with stage IVB disease (*P* = 0.035). Patients with prognostic nutritional index (PNI) ≥ 39.4 had a mPFS of 17.2 months, superior to 10.7 months in those with PNI <39.4 (*P* = 0.030). Regarding safety, Grade 1–2 rash, diarrhea, and oral mucositis were the most common adverse events, with excellent overall tolerability (52).

Lazertinib (trade name: Lazcluze; hereafter referred to as lazertinib) is a third-generation irreversible EGFR-TKI with proven anti-tumor activity against *EGFR*-mutant NSCLC. The integrated analysis of its Phase 1/2 trial (NCT03046992) focused on patients with *EGFR* T790M-positive advanced NSCLC progressing after prior first/second-generation EGFR-TKIs receiving once-daily lazertinib 240 mg. Independent central review confirmed an ORR of 55.3% and a DCR of 89.5%. The mPFS reached 11.1 months, and the median duration of response (mDoR) was 17.7 months.For patients with measurable brain metastases, the intracranial ORR (iORR) was as high as 85.7%, with an intracranial DCR of 100.0%, and the median intracranial progression-free survival was 26.0 months. Lazertinib demonstrated a manageable safety profile; most treatment-emergent adverse events were mild to moderate in severity, with few severe drug-related adverse events observed (53).

### 2.4 The Mechanism of resistance to small molecule inhibitors targeting EGFR

Resistance mechanisms of small−molecule inhibitors targeting EGFR mainly originate from on−target secondary/tertiary mutations and *EGFR* amplification. Secondary T790M mutation accounts for 50%–60% of acquired resistance to first− and second−generation EGFR−TKIs; this mutation impairs drug binding through steric hindrance and increases ATP affinity to induce drug resistance. Tertiary mutations, including C797S, L718Q and G724S, serve as core drivers of resistance to third−generation EGFR−TKI (e.g., Osimertinib). Among these, the C797S mutation occurs in 10%–20% of resistant cases and disrupts the covalent linkage between the drug and EGFR. The cis-configuration of concurrent C797S and T790M confers pan−EGFR−TKI resistance, whereas tumors harboring the trans−configuration remain susceptible to the combination of first− and third−generation EGFR−TKIs. In addition, EGFR amplification or overexpression triggers sustained ligand−independent kinase activation, directly leading to therapeutic resistance (54). Clinical studies indicate that the primary cause of acquired resistance to third-generation EGFR-TKIs is the EGFR tyrosine kinase C797S gene mutation, and EAI045 can specifically inhibit a variety of *EGFR* mutations such as T790M, L858R and L858R/T790M, providing a novel strategy to address resistance to third-generation EGFR-TKIs (55). At this stage, new fourth-generation EGFR-TKIs are still in preclinical development, with the core objective of overcoming acquired resistance mediated by established *EGFR* mutations. These agents aim to refine the NSCLC targeted therapy system, offering new directions and options for the subsequent treatment of *EGFR* mutation-positive NSCLC patients (56).

## 3 Small molecule targeted drugs targeting ALK

*ALK* belongs to the transmembrane RTK family, comprising an extracellular ligand-binding domain, a transmembrane hydrophobic segment and an intracellular tyrosine kinase domain (57, 58). As a highly conserved member of the insulin receptor superfamily, ALK plays an important role in the development of the nervous system and the maintenance of normal physiological homeostasis. Binding of the endogenous ligand ALKAL to ALK’s extracellular domain induces receptor dimerization and autophosphorylation, triggering downstream signaling cascades that regulate cell proliferation, survival and differentiation (59). In 2007, Soda et al. first discovered the echinoderm microtubule-associated protein 4-anaplastic lymphoma kinase fusion gene (*EML4-ALK*) in NSCLC patients (60). The abnormal fusion of *ALK* and *EML4* genes can lead to the continuous abnormal activation of ALK tyrosine kinase, promoting uncontrolled tumor cell proliferation and ultimately contributing to NSCLC pathogenesis. Clinically, NSCLC patients with *EML4-ALK* fusion gene positive are predominantly young, non-smokers or light-smokers, and both *EGFR* and *KRAS* are wild type (61). Therefore, the research and development of targeted drugs against ALK is an important idea for the treatment of NSCLC. At present, the first-generation ALK-TKI Crizotinib (trade name: Xalkori; hereafter referred to as Crizotinib) confers superior clinical outcomes over conventional chemotherapy in clinical practice. However, Crizotinib has limited blood-brain barrier (BBB) penetration ability, and most patients develop resistance within 1–2 years after medication. The second-generation ALK-TKIs Brigatinib (trade name: Alunbrig; hereafter referred to as Brigatinib, Alectinib (trade name: Alecensa; hereafter referred to as Alectinib) and Ceritinib (trade name: Zykadia; hereafter referred to as Ceritinib) have been successively approved for application, which not only significantly improved the therapeutic effect of patients with brain metastasis, but also showed good inhibitory activity against some drug-resistant mutations. Ensartinib (trade name: Beimeina; hereafter referred to as Ensartinib), a class I new drug independently developed in China, also belongs to the second-generation ALK-TKI, providing more treatment options for Chinese patients (62). The third-generation ALK-TKI Lorlatinib (trade name: Lorbrena; hereafter referred to as Lorlatinib) has undergone structural optimization with a more compact molecular conformation and smaller relative molecular mass, and its BBB penetration ability has been further improved. Furthermore, it exhibits high selectivity for both ALK and ROS1, with preclinical evidence supporting efficacy against most ALK drug-resistant mutations (63). The small molecule targeted drugs targeting ALK are shown in **Table 2**.

**Table 2:** Summary of small molecule inhibitors targeting ALK.

Class	Drug	Target	Adaptation disease	Adverse reaction (Grade ≥ 3)
First generation	Crizotinib	ALK	Genetically confirmed target-positive: adult metastatic ALK/ROS1 NSCLC, unresectable/recurrent ALK inflammatory myofibroblastic tumor; ≥ 1 year kids: relapsed ALK-positive anaplastic large cell lymphoma, inoperable inflammatory myofibroblastic tumor (64).	Severe liver injury, fatal ILD, marked QT prolongation, severe bradycardia, severe visual impairment. Children prone to severe cytopenia, GI upset, stomatitis; also fatigue, rash, severe infection, peripheral neuropathy (64).
Second generation	Brigatinib	ALK	First-line for adult ALK+ metastatic NSCLC; or after Crizotinib failure/intolerance (65).	ILD, refractory hypertension, bradycardia, severe eye/liver/pancreas/muscle injury, refractory hyperglycemia and severe GI toxicity; adjust dose or discontinue permanently by grade (65).
	Ceritinib	ALK	For ALK-positive metastatic NSCLC (66).	GI damage, elevated liver/pancreatic enzymes (pancreatitis risk), metabolic and hematologic abnormalities, QT prolongation, pericarditis, interstitial lung disease, lung infection; rare sepsis, myocardial infarction (66).
	Ensartinib	ALK	First-line for adult untreated ALK-positive advanced NSCLC (67).	ILD, severe liver injury, severe rash; elevated transaminase, uric acid, glucose, creatine kinase (CK), decreased lymphocyte and phosphorus; severe fatigue, myalgia, bradycardia, visual disorder (67).
	Alectinib	ALK	Adjuvant for resected ALK+ NSCLC (≥ 4cm or nodal metastasis), first-line advanced setting, second-line post Crizotinib resistance (68).	Severe liver injury, ILD, renal damage, symptomatic bradycardia, elevated CK with severe myalgia, severe hemolytic anemia (68).
Third generation	Lorlatinib	ALK	First-line adult ALK+ metastatic NSCLC or after multiple ALK-TKI resistance (69).	Severe hyperlipidemia, liver injury, central nervous system toxicity, atrioventricular block, ILD, severe hypertension, refractory hyperglycemia (69).
Fourth generation	Neladalkib	ALK	For advanced NSCLC with ALK G1202R/I1171N mutation after Alectinib progression (70).	Only transient transaminase elevation (Grade 3 included) confirmed currently (70).

### 3.1 First-generation ALK inhibitors

First-generation ALK inhibitors are all ATP-competitive agents that abrogate aberrant activation of downstream signaling pathways (such as PI3K-Akt, MAPK, etc.) by competitively binding the ATP binding site of ALK kinase, thereby inhibiting the proliferation, invasion and metastasis of tumor cells (71). Crizotinib, as the representative drug of this class, is an oral small molecule ATP competitive ALK inhibitor that can simultaneously block the signal transduction of cellular-mesenchymal–epithelial transition factor (c-MET) and ROS1. It exhibits high target specificity and potent inhibition of ALK autophosphorylation (72). Updated data from the PROFILE trial series indicated that updated results of the Phase I PROFILE−1001 trial for pretreated patients yielded an ORR of 60.8% and a mPFS of 9.7 months. The Phase II PROFILE−1005 trial reported an ORR of 53% and mPFS of 8.5% for second−or later−line treatment. In the Phase III PROFILE−1007 trial comparing Crizotinib versus chemotherapy in the second−line setting, Crizotinib achieved mPFS of 7.7 months and ORR of 65%, which was significantly superior to chemotherapy mPFS 3.0 months, ORR 20%; [Hazard Ratio (HR) = 0.49, *P*<0.0001]. The Phase III PROFILE−1014 trial evaluated first−line Crizotinib versus platinum plus pemetrexed chemotherapy; patients receiving Crizotinib had mPFS of 10.9 months and ORR of 74%, versus 7.0 months and 45% in the chemotherapy arm (HR = 0.45, *P*<0.001). Among patients with concurrent brain metastases, the 12−week intracranial DCR reached 65% in the Crizotinib group versus 46% in the chemotherapy group (73).

However, Crizotinib has clinical limitations. It is associated with TRAEs, including cytopenia and transaminases. Owing to poor water solubility, high plasma clearance rate and significant first-pass effect, it exhibits a bioavailability of 32% - 66%, which limits its clinical utility (74, 75).

### 3.2 Second-generation ALK inhibitors

Although the first-generation ALK inhibitors improve the prognosis in advanced NSCLC, most patients develop acquired resistance within 1 year of treatment, with brain metastasbeing prevalent. Second-generation ALK inhibitors can target and inhibit secondary drug-resistant mutations in the ALK kinase region (e.g., L1196M, G1269A, etc.), effectively overcoming Crizotinib resistance (71). Key agents in the class include Brigatinib, Ceritinib, Ensartinib, and Alectinib. Compared with the first-generation drugs, they have stronger ALK target affinity, specificity and BBB penetration ability, providing superior control of brain metastases.

Brigatinib is a small−molecule TKI with potent inhibitory activity against ALK. In the global Phase III ALTA−1L trial, previously untreated patients with ALK-positive advanced NSCLC received Brigatinib with a 7-day lead-in of 90mg once daily (QD) followed by escalation to 180 mg (QD). The cohort included 59 Asian and 78 non−Asian patients, with median follow−up durations of 40.7 months for Asian patients and 39.8 months for non−Asian counterparts. Efficacy analyses showed Asian patients achieved a mPFS of 24.0 months, an ORR of 80%, a mDoR of 22.2 months, and an intracranial ORR of 62% among those with baseline brain metastases. Non−Asian patients attained an mPFS of 24.7 months, an ORR of 71%, an mDOR of 41.4 months, and an intracranial ORR of 69% in patients with baseline intracranial lesions. Compared with Crizotinib, brigatinib conferred prominent survival and anti-tumor benefits across both ethnic subgroups, with a favorable OS trend. In terms of safety, Asian patients were predisposed to elevated transaminases, increased creatine phosphokinase (CPK) and constipation (grade ≥ 3 CPK elevation: 34%), whereas non−Asian patients had higher incidences of diarrhea, nausea and peripheral edema (grade ≥ 3 CPK elevation: 21%). The overall incidence of pneumonia was 7% in Asian patients versus 5% in non−Asian patients, with early−onset pneumonia occurring at an identical rate of 3% in both groups. Dose reduction rates were 46% and treatment discontinuation rates 8% among Asian patients, versus 43% and 17% in non−Asian patients, respectively. Pharmacokinetic analyses verified that systemic exposure of Brigatinib was not affected by ethnicity. Collectively, this Phase III trial confirms that first−line Brigatinib yields superior systemic and intracranial efficacy relative to Crizotinib for ALK-positive advanced NSCLC, regardless of ethnicity, with generally comparable safety profiles. This renders Brigatinib a preferred first−line option for Asian patients (76).

Ceritinib is an oral small molecule TKI with multi-targeted activity, selectively inhibiting ALK, insulin-like growth factor-1 receptor (IGF-1R), insulin receptor (InsR) and ROS1, among which the inhibitory effect on the ALK target has high selectivity. It exerts its core anti-tumor effect by blocking ALK autophosphorylation and the phosphorylation of ALK-mediated downstream signaling proteins, thereby abrogating proliferative signaling in *ALK*-positive cancer cells (77, 78). Data from the global phase III ASCEND-4 clinical trial (trial number: NCT01828099) showed that Ceritinib significantly improved PFS compared to pemetrexed combined with platinum chemotherapy regimen in treatment-naive advanced ALK-rearranged NSCLC patients. Confirmed by blinded independent central review (BICR) evaluation, the mPFS of patients in the Ceritinib treatment group was 16.6 months (95% CI:12.6 - 27.2); versus 8.1 months (95% CI: 5.8 - 11.1) in the chemotherapy arm(HR = 0.55;95% CI: 0.42 - 0.73), with an extremely statistically significant difference (*P* < 0.00001). Based on these compelling efficacy data, the FDA officially approved Ceritinib for the treatment of advanced *ALK*-rearranged NSCLC patients in May 2017, with a subsequent indication expansion to include the previously treatment-naive advanced *ALK*-rearranged NSCLC population (79–81).

Ensartinib (Also known as X-396; hereafter referred to as Ensartinib) is a new type of second-generation ALK-TKI with an aminopyridazine-based chemical structure, exhibiting potent activity against central nervous system metastases (82). The results of a phase I/II clinical trial showed that the phase II recommended dose (RP2D) of Ensartinib was set at 225 mg once a day (QD), with preliminary data showing an ORR of 60% and the mPFS was 9.2 months (83). In the Phase III eXalt3 trial, Ensartinib achieved a mPFS of 25.8 months and an ORR of approximately 75% in the intention-to-treat (ITT) population versus Crizotinib in the control arm. Among patients with measurable baseline brain metastases, the intracranial ORR reached 63.6%; the 12-month cumulative incidence of newly developed brain metastases was only 4.2% in patients without baseline intracranial lesions, demonstrating prominent superiority in intracranial disease control. Regarding safety profile, rash and elevated transaminases were the most frequent adverse events, predominantly grade 1–2, with satisfactory overall treatment tolerability. This Phase III trial confirms that first-line Ensartinib provides superior systemic efficacy, remarkable intracranial benefits and well-manageable safety (84).

Among the second-generation ALK small molecule targeted drugs, Alectinib has the best performance in the comprehensive evaluation of three core therapeutic dimensions: safety, efficacy and pharmaceutical properties (85), with a 12-month event-free survival rate of 68.4% (86). The Phase III ALEX trial demonstrated that Alectinib achieved an mPFS of 25.7 months, which was significantly better than 10.4 months of Crizotinib, and could reduce the risk of occurrence and progression of brain metastasis (87, 88).

The TRAEs of second-generation ALK inhibitors are mainly mild to moderate GI toxicities (e.g., nausea, vomiting, etc.) and may also manifest as mild visual disturbances or granulocytopenia, necessitating close clinical monitoring (61). In addition, various drugs have specific TRAEs: the incidence of skin toxicity of Ensartinib is 74.4% (mainly rash and pruritus, of which 10.4% are grade 3) (84); Alectinib has a higher incidence of hepatotoxicity and myalgia (the incidence of myalgia is 28%, mostly grade 1-2, often accompanied by elevated CPK (86).

### 3.3 Third-generation ALK inhibitors

Lorlatinib, a third-generation ALK inhibitor, is a reversible, potent and highly selective dual ALK/ROS1 inhibitor that can overcome a variety of drug-resistant mutations, including G1202R (the most common ALK drug-resistant mutation), and exhibits excellent BBB penetration (89, 90). This renders it a valuable therapeutic option for *ALK* gene-rearranged NSCLC patients (especially drug-resistant and brain metastasis patients) (63). Clinical data showed that the 3-year PFS rate of the Lorlatinib group was 64% (95% CI 55 - 71), which was significantly higher than 19% (95% CI 12 - 27) of the Crizotinib group, and it was superior to Crizotinib in all core efficacy indicators such as ORR, intracranial ORR and time to intracranial progression (91). Its most characteristic TRAEs are hyperlipidemia (incidence rate > 90% in Chinese patients). Lipid levels need to be evaluated before treatment to clarify the need for lipid-lowering intervention, and regular monitoring and simultaneous lipid-lowering treatment are required during treatment. In addition, the incidence of central nervous system toxicity of Lorlatinib (35%) is higher than that of Crizotinib (11%), mainly manifested as cognitive abnormalities, mood changes, etc., mostly grade 1, typically resolve without special intervention, and require dose adjustments in a minority of cases for adequate control, reflecting overall favorable tolerability (92, 93).

### 3.4 Fourth-generation ALK inhibitors

Neladalkib (also known as NVL-655), a rationally designed highly selective fourth-generation ALK inhibitor, exhibits potent preclinical activity against various *ALK* single mutations and Lorlatinib-resistant compound mutations including G1202R, with excellent blood-brain barrier penetration and minimal inhibition of tropomyosin receptor kinase (TRK) to greatly reduce central nervous system toxicity. Its Phase 1/2 ALKOVE-1 trial set the RP2D at 150 mg QD. A total of 253 advanced ALK-positive NSCLC patients progressing after multiple ALK TKI treatments were enrolled, among whom 91% had prior lorlatinib exposure, 19% carried the G1202R resistance mutation and 40% presented with active brain metastases. The overall ORR assessed by BICR was 31% (95% CI: 26% - 37%), accompanied by 12-month and 18-month durable response rates of 64% and 53%. The ORR was 46% (95% CI: 33% - 59%) in 63 Lorlatinib-naïve patients, with 12-month and 18-month durable response rates of 80% and 60%. The G1202R-mutated subgroup achieved an ORR of 68%, and the intracranial ORR was 32% in 92 patients with measurable brain metastases. An exploratory cohort of 44 ALK-TKI-naïve patients showed a first-line ORR of 86% and an intracranial ORR of 78%. Thanks to its unique molecular design that spares TRK binding, Neladalkib has a favorable safety profile, with an adverse event-related treatment discontinuation rate of 5% and a dose reduction rate of 17%. Transaminitis was the most common adverse event, and the majority of cases were reversible (94).

### 3.5 The mechanism of resistance to small molecule inhibitors targeting ALK

Resistance to targeted therapy in ALK-positive NSCLC is mainly categorized into two major types: on-target *ALK* mutations (including single-site mutations such as G1202R, and compound mutations detected in 29% of Lorlatinib-resistant cases), and off-target bypass activation, as well as histological transformation. Four generations of targeted agents have been developed to address this challenge. First-generation Crizotinib is frequently associated with resistance mediated by L1196M mutation and *MET* amplification. Second-generation agents, including Alectinib, Ceritinib, Brigatinib and Ensartinib, fail to overcome the G1202R mutation. Third-generation Lorlatinib exerts potent activity against a single G1202R mutation, but its efficacy is compromised by *ALK* compound mutations and aberrant bypass signaling. The fourth-generation agent NVL-655 remains under clinical investigation, with primary application in patients harboring compound mutations after failure of third-generation TKIs. In cases with bypass resistance, therapeutic strategies include combination therapy with targeted agents against the corresponding signaling pathways, antibody-drug conjugates (ADCs) or platinum plus pemetrexed chemotherapy can be adopted. The ORR of Datopotamab Deruxtecanis 35%, while that of platinum-pemetrexed combination chemotherapy is 29.7%. For patients with oligoprogression, local radiotherapy can be added while maintaining the original targeted therapy. Additionally, circulating tumor DNA (ctDNA) monitoring and combination with dormant cell inhibitors are conducive to delaying the emergence of drug resistance (95).

## 4 Small molecule targeted drugs targeting KRAS

KRAS mutation is one of the most prevalent oncogenic alterations in NSCLC, with a prevalence of 20%-30% in lung adenocarcinoma (96). Located on chromosome 12, *KRAS* was the first discovered proto-oncogene. It’s encoded guanosine triphosphatase (GTPase) as a molecular switch, coupling cell surface growth factor receptors to intracellular signaling pathways and transcription factors to regulate the signal transduction (97, 98). In NSCLC, *KRAS* mutations occur in approximately 25% of cases, predominantly in lung adenocarcinoma, and are positively associated with smoking behavior and distant metastasis in advanced tumors (99). *KRAS* mutation can destroy the intrinsic activity of GTPase, blocking GTPase-activating protein (GAP)-mediated hydrolysis of active guanosine triphosphate (GTP) to inactive guanosine diphosphate (GDP) (97, 98). This aberrant activation sustains EGFR signaling cascades, driving tumor cell proliferation, survival, and progression (99). In addition, *KRAS* mutations are closely linked to resistance to targeted therapies and confer a poor prognosis. They also exhibit distinct ethnic disparities, with a significantly higher prevalence in Caucasians (25% - 50%) than in Asians (5% - 15%) (96, 100). First identified in lung cancer, the most common *KRAS* mutations are Glycine 12 Cysteine (G12C, 41%) and Glycine 12 Valine (G12V, 19%), and NSCLC patients harboring these mutations have shorter PFS (101). Beyond NSCLC (13% of cases), *KRAS* G12C mutations are also detected in 3% of colorectal cancers and 2% of other solid tumors (102). KRAS-targeted small-molecule inhibitors are summarized in **Table 3**.

**Table 3:** Summary of small molecule inhibitors targeting KRAS.

Drug	Target	Adaptation disease	Adverse reaction (Grade ≥ 3)
Sotorasib	KRAS	Single-agent for pretreated advanced NSCLC with FDA-confirmed KRAS G12C mutation (103).	Frequent liver injury, musculoskeletal pain, pneumonia; less diarrhea, dyspnea; elevated transaminase and urine protein in lab tests (103).
Adagrasib	KRAS	Single-agent for pretreated KRAS G12C-mutant advanced NSCLC; combined with cetuximab for late-line colorectal cancer (104).	Common pneumonia, dyspnea, liver injury, fatigue, QT prolongation, bone pain; lab findings: lymphopenia, anemia, hyponatremia and elevated transaminase (104).

At present, international multicenter clinical trials have confirmed that Sotorasib (trade name: Lumakras; hereafter referred to as Sotorasib) and Adagrasib (trade name: Krazati; hereafter referred to as Adagrasib) can significantly improve the clinical prognosis of patients with advanced *KRAS-*G12C mutant NSCLC, providing a novel therapeutic option for this group of patients (105, 106). As the first-in-class KRAS-targeted agent, Sotorasib forms an irreversible covalent bond with the unique cysteine of *KRAS* G12C (resulting from glycine). It locks the mutant KRAS protein in an inactive state, thereby blocking downstream signal transduction, and has no inhibitory activity against wild-type KRAS. This agent selectively inhibits KRAS-mediated signaling in *KRAS* G12C-positive tumor cell lines, suppressing proliferation and inducing apoptosis (107). Clinical studies demonstrate that in pretreated advanced NSCLC patients with KRAS p.G12C mutation, the ORR of Sotorasib is 37.1%, the mDoR is 11.1 months, the mPFS is 6.8 months, and the mOS is 12.5 months (108). Regarding TRAEs of Sotorasib, the most frequent adverse events are concentrated in the gastrointestinal and hepatobiliary systems. Gastrointestinal toxicities are dominated by diarrhoea [196 cases, reporting odds ratio (ROR) = 5.54], nausea and vomiting; hepatobiliary toxicity yields an extremely robust safety signal, with a total of 55 hepatotoxicity reports (ROR = 39.18), and elevated transaminases [43 cases each for aspartate aminotransferase (AST) and alanine aminotransferase (ALT)] as the hallmark laboratory abnormality. Severe toxicities validated via four signal detection algorithms include hepatic failure (9 cases, ROR = 7.61), drug-induced liver injury (12 cases, ROR = 5.92) and pancreatitis (10 cases, ROR = 5.10); pulmonary embolism, with merely 19 reported cases, represents a relatively rare serious adverse reaction (109).

Adagrasib is a small molecule covalent *KRAS* G12C inhibitor that selectively binds to and locks *KRAS* G12C in the inactive GDP-bound state (110). The phase III clinical trial (KRYSTAL-12 study, NCT04685135) compared the efficacy of Adagrasib and Docetaxel in the treatment of previously treated patients with locally advanced or metastatic NSCLC with *KRAS* G12C mutation. The results showed that Adagrasib significantly improved mPFS compared with the docetaxel group (5.49 months vs. 3.84 months, HR = 0.58, 95% CI: 0.45 - 0.76, *P <* 0.0001), with comparable safety profiles between groups (111). Adagrasib-related TRAEs are mainly gastrointestinal toxicities and metabolic/nutritional disorders, with prolonged electrocardiogram QT interval identified as a positive cardiac safety signal. The predominant gastrointestinal manifestations include nausea (42 cases, ROR = 4.24), diarrhoea (41 cases, ROR = 4.49) and vomiting (30 cases, ROR = 5.36), while decreased appetite (15 cases, ROR = 4.43) and dehydration (11 cases, ROR = 7.35) stand out as prominent metabolic adverse events. Serious neurological TRAEs such as seizure are rare (109).

MRTX1133 is a selective, non-covalent small-molecule *KRAS* G12D inhibitor developed by Mirati Therapeutics. The oncogenic *KRAS* G12D mutation is prevalent in both pancreatic ductal adenocarcinoma (PDAC) and NSCLC, with striking differences in mutational landscape between the two tumor types. Statistically, *KRAS* G12D mutations account for 41.8% of all *KRAS* alterations in PDAC, while the incidence of *KRAS* G12C mutations is lower than 2%. In contrast, NSCLC is dominated by *KRAS* G12C mutations with a prevalence of approximately 13%, and the targeted agent sotorasib has been clinically approved for *KRAS* G12C-mutant NSCLC. However, there are currently no approved targeted therapies for *KRAS* G12D-driven NSCLC, leaving a critical unmet clinical gap in the treatment of this patient subgroup. Preclinical *in vitro* assays have confirmed that MRTX1133 binds potently to inactive KRAS G12D protein, with a half-maximal inhibitory concentration (IC_50_) below 2 nM, and suppresses the proliferation of *KRAS* G12D-mutant tumor cells at a median IC_50_ of approximately 5 nM. Notably, MRTX1133 exhibits no inhibitory effect on *KRAS* G12C-mutant lung cancer cells, demonstrating its high mutation selectivity. *In vivo* evaluations using *KRAS* G12D genetically engineered lung cancer mouse models have shown that MRTX1133 treatment induces robust tumor regression and promotes tumor cell apoptosis. Mechanistically, MRTX1133 enhances the intratumoral infiltration of CD8+ T cells in lung tumor tissues, and combinatorial treatment with immune checkpoint inhibitors exerts potent synergistic anti-tumor effects. Despite these promising preclinical efficacy profiles, MRTX1133 possesses inherent therapeutic limitations. In preclinical models, the single-agent anti-tumor activity of MRTX1133 in lung cancer xenografts is inferior to that observed in PDAC models. Furthermore, compensatory activation of the EGFR and YAP signaling pathways drives the development of intrinsic and acquired resistance to MRTX1133, thereby compromising its therapeutic efficacy. The phase I/II clinical trial (NCT05737706), initiated in 2023, recruits patients with advanced *KRAS* G12D-mutant malignancies, including NSCLC, PDAC, and colorectal cancer. This trial is still ongoing, and no mature clinical response data for the NSCLC cohort has been published to date (112). Overall, the development of KRAS G12D-targeted small-molecule inhibitors offers novel insights and promising therapeutic candidates for the clinical treatment of *KRAS* G12D-mutant NSCLC.

Resistance to KRAS-targeted agents is divided into two categories: genetic mutations and non-genetic mechanisms. Genetic resistance accounts for approximately 50%–60% of all resistance cases. On-target resistance includes secondary *KRAS* point mutations (detected in 20%–25% of resistant patients) and *KRAS* amplification (observed in around 7% of Sotorasib-resistant cases). Bypass signaling abnormalities contribute to 35%–45% of genetic resistance: downstream mutations (e.g., *BRAF*, *PIK3CA*) account for roughly 15% of resistant NSCLC, while deletion of tumor suppressor genes (e.g., *KEAP1*, *PTEN*) is identified in an additional 12%. Non-genetic resistance constitutes 40%–50% of total resistance, driven primarily by compensatory activation [*GFR*, neuroblastoma RAS viral oncogene homolog (*NRAS*), etc] following drug exposure (more than 30% of non-genetic resistance), YAP-mediated epithelial-mesenchymal transition (EMT) (present in 18% of resistant specimens), HDAC5-regulated macrophage infiltration and stromal remodeling (stroma-related resistance reaches 30% in pancreatic cancer), MDR1-mediated drug efflux (approximately 9% of related resistance), and the formation of dormant drug-tolerant persister (DTP) cells (10% - 15% of non-genetic resistance). The coexistence of multiple resistance mechanisms accelerates treatment failure with single-agent therapy (113).

## 5 Small molecule targeted drugs targeting ROS1

*ROS1* is an oncogene located at chromosome 6q22.1 that encodes a member of the insulin receptor subfamily. *ROS1* fusion genes have been detected across multiple tumor types, with the HLA class II histocompatibility antigen gamma chain-c-ros oncogene 1 receptor tyrosine kinase (*CD74-ROS1*) fusion is the most common, accounting for 44%, followed by ezrin-c-ros oncogene 1 receptor tyrosine kinase(*EZR-ROS1*), syndecan 4-c-ros oncogene 1 receptor tyrosine kinase (*SDC4-ROS1*), and solute carrier family 34 member 2-c-ros oncogene 1 receptor tyrosine kinase (*SLC34A2-ROS1*) fusion types (114–116). *ROS1* fusion proteins drive cell proliferation, activation, and cell cycle progression by activating downstream signaling pathways, thereby accelerating the occurrence and development of NSCLC. Clinical evidence indicates that *ROS1* rearrangement occurs in 1% - 2% of NSCLC patients, predominantly in non-squamous subtypes. Notably, 36% of ROS1-positive NSCLC patients are simultaneously complicated with other oncogenic abnormalities, including *EGFR* mutation, *KRAS* mutation, *MET* amplification, or *ALK* translocation (117). The central nervous system is a common metastatic site of *ROS1* fusion-positive NSCLC, about 36% of patients have brain metastasis at initial diagnosis, and the remaining patients may develop intracranial metastasis during follow-up. Therefore, the treatment strategy for ROS1-positive NSCLC needs to take into account the involvement of the central nervous system, and it is recommended to routinely carry out ROS1 detection for all NSCLC patients with brain metastasis (118). The summary of small molecule inhibitors targeting ROS1 is shown in **Table 4**.

**Table 4:** Summary of small molecule inhibitors targeting ROS1.

Class	Drug	Target	Adaptation disease	Adverse reaction (Grade ≥ 3)
First generation	Crizotinib	ROS1	Genetically confirmed target-positive: adult metastatic ALK/ROS1 NSCLC, unresectable/recurrent ALK inflammatory myofibroblastic tumor; ≥ 1 years kids: relapsed ALK-positive anaplastic large cell lymphoma, inoperable inflammatory myofibroblastic tumor (64).	Severe liver injury, fatal ILD, marked QT prolongation, severe bradycardia, severe visual impairment. Children prone to severe cytopenia, GI upset, stomatitis; also fatigue, rash, severe infection, peripheral neuropathy (64).
Second generation	Entrectinib	ROS1	Adult ROS1-positive metastatic NSCLC and NTRK-fusion advanced solid tumors in patients ≥ 1 month old (119).	Heart failure, QT prolongation, elevated liver enzymes, severe hyperuricemia, pathological fracture, severe neurologic, GI, respiratory and ocular toxicity (119).
	Brigatinib	ROS1	First-line treatment for previously untreated ROS1-positive NSCLC (120).	ILD, refractory hypertension, bradycardia, severe eye/liver/pancreas/muscle injury, refractory hyperglycemia and severe GI toxicity; adjust dose or discontinue permanently by grade (65).
	Repotrectinib	ROS1	Adult ROS1-positive advanced NSCLC and NTRK-fusion advanced solid tumors in patients ≥ 12 years old (121).	Severe neurotoxicity, ILD, elevated liver/muscle enzymes, severe hyperuricemia, pathological fracture, marked fatigue plus respiratory and GI adverse events (121).
	Taletrectinib	ROS1	For adult locally advanced/metastatic ROS1-fusion positive NSCLC (122).	Clinical: pneumonia, pleural effusion, liver injury, QT prolongation, Grade 3 fracture; lab abnormalities: elevated ALT/AST, neutropenia, elevated CK, lymphopenia, decreased hemoglobin (122).
Third generation	Lorlatinib	ROS1	For advanced metastatic ALK/ROS1-fusion positive NSCLC (123).	Dyslipidemia, severe elevated liver enzymes, serious central neuropathy, Grade ≥ 3 hypertension and atrioventricular block, severe ILD, severe hyperglycemia, severe edema, peripheral neuropathy, fatigue and multisystem injuries (69).

### 5.1 First-generation ROS1 inhibitors

Sequence homology within the ATP-binding site between ROS1 and ALK reaches 77%, and this structural similarity enables multiple ALK-TKIs to exhibit inhibitory activity against ROS1. Among these, Crizotinib and Lorlatinib (trade name: Lorbrena; hereafter referred to as Lorlatinib) have been clinically validated (124). A clinical study led by Alice T. Shaw et al. investigating crizotinib in 50 patients with ROS1-rearranged advanced NSCLC reported 3 complete responses, an ORR of 72%, and a mPFS of 19.2 months (125). The updated results of the PROFILE 1001 trial led by Shaw et al., with a median follow-up of 62.6 months, enrolled 53 heavily pretreated advanced NSCLC patients harboring *ROS1* fusions. All patients received Crizotinib 250 mg twice daily (BID) orally, achieving an ORR of 72%, a mDoR of 24.7 months, a mPFS of 19.3 months, and a mOS of 51.4 months (4-year survival rate: 51%). Notably, *ROS1* fusion subtype did not impact treatment efficacy or survival outcomes (118). Since the first discovery of *ROS1* rearrangement in NSCLC, new targeted drugs have continued to emerge; however, Crizotinib remains the preferred first-line therapy for *ROS1*-rearranged NSCLC (114). A critical limitation of crizotinib is that the central nervous system acts as the initial progression site in nearly half of treated patients; specifically, 46% of progressive cases show isolated central nervous system recurrence without extracranial lesions, which can be due to the drug’s poor BBB penetration (126). Common TRAEs of Crizotinib (incidence ≥ 25%) include visual disturbances, nausea, diarrhea, vomiting, edema, and constipation, mostly grade 1~2. Clinically, close monitoring for severe TRAEs such as hepatotoxicity and interstitial pneumonia is warranted (127).

### 5.2 Second-generation ROS1 inhibitors

Entrectinib is a potent, selective second-generation TKI targeting *ROS1*, neurotrophic tyrosine kinase eceptor 1/2/3 (*NTRK1/2/3*), and *ALK* fusion mutations. It demonstrates superior anti-tumor activity and BBB penetration compared to Crizotinib, making it particularly suitable for *ROS1* fusion-positive patients with intracranial metastases (128, 129). A 2020 pooled analysis published in The Lancet Oncology integrated data from two Phase I trials (ALKA-372-001, STARTRK-1) and the global Phase II basket trial STARTRK-2, enrolling treatment-naive patients with locally advanced or metastatic *ROS1*-fusion-positive NSCLC. Among the 53 evaluable patients receiving Entrectinib, the ORR was 77% (95% CI: 64% - 88%), with a mDoR of 24.6 months and a mPFS of 19.0 months. In 20 patients with baseline brain metastases assessed by BICR, the intracranial ORR was 55% (95% CI: 32% - 77%) and the median intracranial duration of response reached 12.9 months. In the safety population (n = 134), 59% experienced only grade 1–2 TRAEs, 34% developed grade 3–4 TRAEs (most commonly weight gain and neutropenia), and severe adverse events occurred in 11% of patients; no treatment-related deaths were reported. Overall, Entrectinib exhibited a favorable safety profile and manageable tolerability (130). On August 15, 2022, China’s National Medical Products Administration (NMPA) officially approved Entrectinib for adult patients with locally advanced or metastatic NSCLC with *ROS1* fusion positive, leading to its upgrade to a GradeI recommendation in the 2023 Chinese Society of Clinical Oncology guidelines. Its most common TRAEs are mainly fatigue (46%), dysgeusia (42%), and paresthesia (29%), mostly mild and well tolerated (131).

Beyond Entrectinib, several ROS1 inhibitors are currently in clinical research or application, including Repotrectinib (trade name: Augtyro; hereafter referred to as Repotrectinib) and Brigatinib. Repotrectinib is a new generation of multi-target inhibitor of ROS1/TRK/ALK, with preclinical studies confirming its significant inhibitory activity on *ROS1* G2032 mutation (132). Interim results from the ongoing global phase I/II clinical trial showed that among 71 patients receiving first-line Potrectinib treatment, the ORR reached 79%, the mPFS was 34.1 months, and the mOS was 35.7 months. In 56 patients with prior TKI exposure, the ORR was 38%, the mPFS was 14.8 months, and the OS was 9.0 months. In addition,17 patients with ROS1 glycine 2032 arginine (*ROS1* G2032R) mutation achieved an ORR of 59%, with robust intracranial efficacy across all subgroups. The TRAEs of Repotrectinib are mainly grade 1 - 2, including dizziness (62%), fatigue (39%), constipation (33%), dysgeusia (33%) and dyspnea (28%), without grade 3–4 TRAEs, with better safety and easy management. Its favorable safety profile and ease of management support promising application in *ROS1*-rearranged NSCLC (133). Brigatinib is a dual ROS1/ALK inhibitor with additional activity against *EGFR* mutations, approved in 2017 for the second-line treatment of ALK-positive NSCLC. A clinical study of Brigatinib involving 8 patients with *ROS1*-rearranged NSCLC showed that the Crizotinib-naive single patient achieved a mPFS of 21.6 months; the ORR of 7 patients with Crizotinib resistance was 29%, of which 1 patient (14%) had stable disease, and 4 patients had mPFS of 7.6, 2.9, 2.0 and 0.4 months, respectively. These data suggested that Brigatinib has a modest clinical efficacy in patients with *ROS1*-rearranged NSCLC with Crizotinib resistance (134). *In vitro* experiments confirmed that Brigatinib, Crizotinib and Ceritinib can all significantly inhibit the proliferation of Ba/F3 cells; however, clinical trials in ALK-positive NSCLC reported higher rates of grade 3–5 TRAEs with Brigatinib compared to Crizotinib, predominantly GI symptoms (135).

Taletrectinib (trade name: Ibtrozi; hereafter referred to as Taletrectinib) is an oral selective tyrosine kinase inhibitor targeting ROS1. Preclinical data demonstrated that potent inhibition of wild-type *ROS1* and the G2032R resistance mutation, with excellent BBB penetration and low Tropomyosin receptor kinase B (TRKB), substantially reduces central nervous system adverse reactions. A Phase I study conducted in the United States and Japan established a maximum tolerated dose (MTD) of 800 mg once daily (QD). In ROS1-TKI-naive patients, the ORR was 66.7% with a mPFS of 29.1 months, while the ORR was 33.3% in Crizotinib-resistant patients. The domestic Phase I/II TRUST trial adopted a 600 mg QD dose of Taletrectinib: treatment-naive patients had an ORR of 92.5%, 52.6% in Crizotinib-pretreated patients, and the intracranial ORR was 91.7% among patients with brain metastases. Elevated transaminases and GI discomfort were the most common adverse events, with severe dizziness rarely reported. Taletrectinib has been granted the FDA Breakthrough Therapy Designation. Interim results from the global multicenter Phase II TRUST-II trial showed an ORR of 94% and a DCR of 100% in treatment-naive patients. For patients with prior TKI exposure, the ORR was 55%, and the DCR was 91%, with an overall manageable safety profile (136).

### 5.3 Third-generation ROS1 inhibitors

Lorlatinib is a CNS-penetrant third-generation TKI with highly selective kinase inhibitory activity against ALK and ROS1. Compared to Crizotinib and Ceritinib, Lorlatinib exhibits more potent ROS1-targeted activity (124, 137). The results of a phase II clinical trial involving 47 patients with *ROS1* gene-rearranged NSCLC showed that treatment-naive patients achieved an ORR of 61% and an mPFS of 21.0 months, while Crizotinib-pretreated patients had an ORR of 26% and the mPFS was 8.5 months. Notably, among 6 treatment-naive patients with brain metastasis, Lorlatinib achieved an iORR of 67%, reflecting its therapeutic advantages in central nervous system metastatic lesions (138). At present, Lorlatinib has been recommended by clinical guidelines as a post-line TKI treatment drug for patients with *ROS1* fusion-positive lung cancer. Preclinical studies confirmed sustained sensitivity to specific resistance mutations arising from early ROS1 TKI treatment (e.g., *ROS1* S1986F/Y) with IC_50_ maintained at 1.0 - 1.6 nmol/L (124). However, it lacks inhibitory activity against two key mutations: *ROS1* G2032R (139)and L2026M (124, 140). In addition, secondary mutations in the *ROS1* gene were detected in 46% of progressive tumor samples from Lorlatinib-treated *ROS1*-rearranged NSCLC patients, with G2032R being the most prevalent (32%), followed by L2086F (11%). These findings provide critical guidance for treatment adjustments in refractory patients (141). Lorlatinib-related TRAEs are diverse, mainly dominated by metabolic reactions, neurological reactions, psychiatric reactions and oedema-related reactions. Most TRAEs occur within the first two months of Lorlatinib treatment initiation with a median onset time of 51 days, while 11.81% of adverse events emerge after one year of medication, showing variable onset times across different treatment periods. Several novel unlabelled TRAEs not mentioned in the package insert were newly detected, including pulmonary arterial hypertension, radiation necrosis and pericardial effusion. A total of 25.98% of adverse event reports recorded death as a serious adverse outcome; the majority of remaining adverse reactions could be relieved and improved with drug discontinuation, dose reduction, or supportive symptomatic management (142).

### 5.4 The mechanism of resistance to small molecule inhibitors targeting ROS1

Resistance to targeted therapy in ROS1-positive NSCLC is mainly mediated by four categories of mechanisms. On-target kinase mutations account for 50%–60% of all resistance cases, with G2032R mutation is the most prevalent and renders Crizotinib and Entrectinib ineffective. For patients harboring this mutation, the Repotrectinib and Taletrectinib achieve an ORR of 59% and 57.1%, respectively. The L2086F mutation confers resistance to all Type I ROS1-TKIs, with Cabozantinib achieving an ORR of approximately 30% in small cohorts. Rare mutations such as D2033N and S1986F can be treated with Entrectinib or Repotrectinib as appropriate. Bypass pathway activation contributes to 20%–30% of resistance, with *MET* amplification being the most common. Combination therapy of ROS1-TKIs with Capmatinib or Savolitinib yields an ORR of 42%–45%. For cases with concurrent *KRAS* G12C alterations, addition of Sotorasib achieves an ORR of nearly 33%, and Osimertinib is used for aberrant EGFR signaling. Histological transformation from adenocarcinoma to small cell lung cancer or squamous cell carcinoma contributes to 2%–5% of resistance. After discontinuation of targeted agents, the EP (Etoposide + Cisplatin) with or without immunotherapy is recommended, achieving an ORR of 40%–55%. The remaining 10%–15% of cases have no identifiable resistance drivers. Platinum-based chemotherapy combined with anti-angiogenic agents is preferred (ORR: 25%–35%), whereas single-agent immunotherapy only achieves an ORR of 13%–17%. Currently, ADCs targeting ROS1 are still under clinical investigation (143). A preclinical drug susceptibility assay using Ba/F3 cells engineered to express CD74-ROS1. In Crizotinib-acquired resistance, secondary mutations within the ROS1 kinase domain account for 50%–60% of cases. The G2032R mutation was the most prevalent (41%), followed by D2033N, S1986Y/F, L2026M and L1951R. *In vitro* results demonstrated that Crizotinib and Entrectinib were only active against wild-type ROS1. Ceritinib and Brigatinib inhibited only L2026M. Lorlatinib showed efficacy against D2033N, S1986Y/F and L2026M, but failed to inhibit G2032R and L1951R. Next-generation candidate agents such as Taletrectinib exhibited potent *in vitro* activity against most resistance mutations, including G2032R. The remaining 40%–50% of resistance is driven by bypass pathway activation or EMT. For resistance caused by activated EGFR, KIT or KRAS bypass pathways, combination therapy of ROS1 inhibitors with corresponding targeted agents is required. Single-agent targeted therapy shows no efficacy against EMT-mediated resistance, while pemetrexed presents definite anti-tumor activity *in vitro* (144).

## 6 Small molecule targeted drugs targeting BRAF

The BRAF protein encoded by the *BRAF* gene belongs to the RAF kinase family and serves as a key regulatory molecule in the MAPK signal cascade (145). Upon RAS activation, BRAF subsequently activates mitogen-activated protein kinase (MEK), which phosphorylates cytoplasmic extracellular signal-regulated kinase1/2 (ERK1/ERK2). Phosphorylated extracellular signal-regulated kinase (ERK) translocates to the nucleus, where it phosphorylates and activates multiple transcription factors to regulate cell apoptosis, proliferation, and migration while upregulating the expression of oncogenes involved in carcinogenesis (146–149). In lung adenocarcinoma NSCLC patients, the incidence of *BRAF* gene mutation is about 2%-4%, with over 50% concentrated at codon 600 of exon 15 in the kinase domain, showing the substitution of valine with glutamic acid [valine 600 glutamic acid (V600E)] (150). *BRAF* Val600Glu (*BRAF* V600E) mutation renders BRAF kinase independent of RAS regulation, leading to constitutive activation and sustained downstream signaling through ERK, which drives tumor progression (151). Clinical characteristics reveal higher *BRAF* V600E mutation rates in women, never-smokers and elderly individuals (152), while non-V600E *BRAF* mutations are more prevalent in men and current or former smokers (152, 153). The cornerstone therapeutic strategy for *BRAF* V600E-mutant NSCLC involves the core strategy is to block both BRAF and its downstream MEK signal molecules, with clinical practice favoring combinations of BRAF inhibitor and MEK inhibitor (154). The summary of small molecule inhibitors targeting BRAF is shown in **Table 5**.

**Table 5:** Summary of small molecule inhibitors targeting BRAF.

Drug	Target	Adaptation disease	Adverse reaction (Grade ≥ 3)
Dabrafenib	BRAF	Monotherapy for advanced BRAF V600E-mutant melanoma; combination with Trametinib for multiple malignancies harboring BRAF V600E/K mutations (155).	May induce new primary malignancies, severe fever with shock, severe hemorrhage, cardiac, ocular and cutaneous injuries; severe adverse reactions including severe hemolysis, diabetic ketoacidosis and hemophagocytic syndrome may also occur (155).
Encorafenib	BRAF	Indicated for BRAF-mutant advanced melanoma and colorectal cancer (156).	Predominantly severe cutaneous adverse reactions, accompanied by elevated liver enzymes, severe hyperglycemia, hemorrhage, venous thrombosis and severe ILD (156).

### 6.1 Dabrafenib combined with trametinib

Dabrafenib (trade name: Tafinlar; hereafter referred to as Dabrafenib) selectively inhibits mutant BRAF kinase, while Trametinib (trade name: Mekinist; hereafter referred to as Trametini) reversibly and selectively inhibits MEK1/2 activation and kinase activity via allosteric regulation. Their combination synergistically blocks aberrant MAPK signaling pathway activation, suppressing proliferation and survival of *BRAF* V600 mutant tumor cells with superior efficacy compared to monotherapy (157).

This combination is designated a Grade I recommendation as the preferred first-line therapy for advanced *BRAF* V600E-mutant NSCLC in the 2023 Chinese Society of Clinical Oncology (CSCO) Guidelines (158). Preclinical studies confirm that dual BRAF/MEK inhibition abrogates paradoxical MAPK pathway hyperactivation triggered by BRAF monotherapy, alleviates drug-induced epidermal proliferative lesions in rodent models, and confers superior anti-tumor potency against *BRAF*-mutant xenografts relative to single-agent regimens, while sustaining prolonged suppression of tumor proliferation (159). Data from the Phase II BRF113928 trial (n = 93) demonstrate that patients receiving Dabrafenib 150 mg BID plus trametinib 2 mg QD achieved robust outcomes: first-line treatment-naive patients(n = 36) had an ORR of 63.9% (95% CI: 46.2% - 79.2%), mPFS of 14.6 months, and mOS of 24.6 months (5-year survival rate: 25%); pretreated patients (n = 57) had an ORR of 68.4% (95% CI: 55.3% - 79.5%), mPFS of 10.2 months, and mOS of 18.2 months (5-year survival rate: 21%) (160). Consistent findings from the Phase II NCT01336634 trial show first-line ORR of 64% (95% CI: 46% - 79%) and mPFS of 10.9 months (95% CI: 7.0 - 16.6 months). The most common TRAEs include fever (46%, signature TRAE) and GI toxicities, with Grade ≥ 3 fever occurring in 11% of patients and prone to recurrence during treatment (150).

### 6.2 Encorafenib combined with binimetinib

Encorafenib (trade name: Braftovi; hereafter referred to as Encorafenib) is an oral small-molecule BRAF kinase inhibitor targeting the MAPK signaling pathway. Binimetinib (trade name: Mektovi; hereafter referred to as Binimetinib) is an oral small-molecule MEK inhibitor that acts on the core RAS-RAF-MEK-ERK signaling pathway; blockade of this pathway suppresses tumor cell proliferation (161). This dual-target combination has demonstrated efficacy and favorable safety in Phase II trials for advanced *BRAF* V600-mutant NSCLC (treatment-naïve and pretreated populations) and is classified as a grade III recommendation in the CSCO Guidelines. The PHAROS study (NCT03915951) specifically evaluated Encorafenib combined with Binimetinib in *BRAF* V600E-mutant advanced NSCLC: first-line patients achieved an ORR of 75% (95% CI: 62%-85%) with mPFS not reached (95% CI: 15.7 months-not reached); while pretreated patients had an ORR of 46% (95% CI: 30% - 63%) and the mPFS of 9.3 months (95% CI: 6.2 months-not reached) (162). The most common TRAEs are nausea, diarrhea and fatigue, with an incidence rate of more than 30% (4).

### 6.3 The mechanism of resistance to small molecule inhibitors targeting BRAF

Resistance to combined BRAF and MEK inhibitor therapy in *BRAF* V600E-mutant NSCLC is primarily mediated by four mechanisms. First, reactivation of the MAPK pathway accounting for 40%–50% of resistance cases, is driven by oncogenic alterations in *KRAS*, *NRAS*, *MEK* and *ERK*, as well as *BRAF* splicing variants. For tumors harboring *KRAS* G12C mutations, combining KRAS inhibitors with the original dual *BRAF/MEK* regimen achieves an ORR of 28%–35%. ERK inhibitors and novel dimer-selective BRAF inhibitors are under active clinical investigation, with early-phase data supporting their potential in reversing MAPK reactivation-mediated resistance. Second, secondary *BRAF* mutations or *BRAF* gene amplification occur in approximately 15% of cases, resulting in resistance to prior targeted therapies. The standard of care for this subgroup is chemotherapy plus immunotherapy, which yields an ORR of 20%–30%. Third, aberrant activation of bypass pathways, including EGFR, MET, PI3K/AKT, and FGFR, contributes to 30%–35% of resistance. For *EGFR* abnormalities, the triple combination of Osimertinib plus BRAF and MEK inhibitors is the preferred, with an ORR of 45%–58%. For *MET* amplification, Savolitinib combined with the dual targeted therapy achieves an ORR of 35%–40%. Alpelisib can be added for patients with *PIK3CA* mutations. Fourth, histological transformation from adenocarcinoma to small cell lung cancer and other subtypes is observed in 5%–8% of resistant cases. Targeted therapy should be discontinued, and the Etoposide + Cisplatin regimen chemotherapy with or without immunotherapy is administered, with an ORR of approximately 52%. Additionally, about 20% of patients present primary resistance due to baseline co-mutations such as *KRAS* and *TP53*. For this population, first-line chemotherapy plus immunotherapy is the preferred strategy, as dual BRAF/MEK inhibition alone confers minimal clinical benefit (163).

## 7 Small molecule targeted drugs targeting TRK

*NTRK* gene fusion serves as a key oncogenic driver across multiple tumors but is rare in NSCLC (incidence < 1%) (164). The *NTRK* gene family comprises *NTRK1, NTRK2, and NTRK3*, which encode neurotrophic tyrosine kinase receptor type 1 (TRKA), neurotrophic tyrosine kinase receptor type 2 (TRKB), and encode neurotrophic tyrosine kinase receptor type 3 (TRKC) receptors, respectively. These receptors are highly expressed in neuronal tissues and regulate cell proliferation, differentiation and apoptosis by binding to neurotrophin ligands, maintaining the normal nervous system development and function (165, 166). TRK receptors share a canonical structure, specifically including an extracellular ligand-binding domain (containing immunoglobulin-like regions and leucine-rich motifs), a transmembrane domain and an intracellular tyrosine kinase domain. Ligand binding induces receptor dimerization and tyrosine kinase autophosphorylation, activating different downstream signaling pathways: the NGF/TRKA pathway activates the RAS/MAPK signal axis, the BDNF/NT-4/TRKB pathway activates the RAS/ERK and PLC-γ pathways, and the NT-3/TRKC pathway preferentially activates the PI3K/AKT pathway. The above pathways mediate the pro-proliferative and anti-apoptotic effects of cells, respectively (166–169). Among *NTRK* oncogenic mechanisms, gene fusions are the most clinically relevant. These arise from chromosomal rearrangements fusing the TRK kinase domain and partner genes [e.g., ETS variant transcription factor 6 (*ETV6*), Tropomyosin 3 (*TPM3*), Lamin A/C (*LMNA*)] through chromosomal rearrangement, and ultimately lead to ligand-independent dimerization and continuous signal activation of the receptor (165). Intrachromosomal or interchromosomal rearrangement connects the 3’ end sequence of *NTRK1, NTRK2*, or *NTRK3* with the 5’ end sequence of other genes, and the formed fusion product is an oncoprotein. This oncoprotein can lead to ligand-independent constitutive activation of TRK kinase and trigger abnormal TRK signal transduction through dimerization, ultimately promoting cancer cell transformation, proliferation, migration and invasion. Notably, not all fusion partner genes contain canonical dimerization domains, and the underlying mechanism of TRK activation in these cases warrants further investigation. The summary of small molecule inhibitors targeting TRK is shown in **Table 6**.

**Table 6:** Summary of small molecule inhibitors targeting TRK.

Class	Drug	Target	Adaptation disease	Adverse reaction (Grade ≥ 3)
First generation	Larotrectinib	TRK	For NTRK-fusion advanced solid tumors across all age groups without resistant mutations; companion diagnostic testing required prior to use (170).	Severe liver injury, neurological abnormalities, hematologic and electrolyte disorders, severe systemic, gastrointestinal and skeletal adverse reactions; dose reduction is required for severe cases, and permanent discontinuation upon recurrent intolerance (170).
	Entrectinib	TRK	Adult ROS1-positive metastatic NSCLC and NTRK-fusion advanced solid tumors in patients ≥ 1 month old (119).	Heart failure, QT prolongation, elevated liver enzymes, severe hyperuricemia, pathological fracture, severe neurologic, GI, respiratory and ocular toxicity (119).
Second generation	Repotrectinib	TRK	Adult ROS1-positive advanced NSCLC and NTRK-fusion advanced solid tumors in patients ≥ 12 years old (121).	Severe neurotoxicity, ILD, elevated liver/muscle enzymes, severe hyperuricemia, pathological fracture, marked fatigue plus respiratory and GI adverse events (121).
	Zurletrectinib	TRK	Indicated for NTRK-fusion solid tumors and disease progression after first-generation TRK inhibitor therapy (predominantly single-point mutations), prioritized for patients with brain metastases (171).	/

### 7.1 First-generation TRK inhibitors

#### 7.1.1 Larotrectinib

Larotrectinib (trade name: Vitrakv; hereafter referred to as Larotrectinib) is a highly selective ATP-competitive inhibitor designed by structural biology methods. Enzymatic assays demonstrate its IC_50_ of 5.3~11.5 nmol/L for TRKA, TRKB, and TRKC, while the cellular IC_50_ levels are 9.8~25 nmol/L (172). Its main mechanism involves competitive binding to the ATP-binding sites of intracellular TRKA/B/C, inhibiting the catalytic activity and autophosphorylation process of TRK, blocking the conduction of abnormal downstream signaling pathways, and ultimately exerting a specific anti-tumor effect (173). A phase I dose-escalation study reported by Hong et al. reported a 100% ORR in 8 BICR-confirmed *NTRK* fusion-positive solid tumors patients, including 2 cases of complete response (CR) and 6 cases of partial response (PR) (174). Pooled data from 3 subsequent phase I/II clinical studies (NCT02637687, NCT02576431, NCT02122913) in 153 evaluable patients showed an ORR of 79% (16% CR, 63% PR), with median DoR, PFS, and OS of patients were 35.2 months, 28.3 months, and 44.4 months, respectively (175). The phase I adult dose-escalation trial LOXO-TRK14001 established the recommended oral dose of Larotrectinib as 100 mg twice daily. A total of 55 patients with advanced solid tumours harbouring *NTRK1/2/3* fusions confirmed by next-generation sequencing (NGS) or fluorescence *in situ* hybridization (FISH) were enrolled, consisting mostly of adults with a small subset of older adolescents. *ETV6-NTRK3* and *TPM3-NTRK1* were the predominant fusion variants, and the enrolled tumour types included salivary gland carcinoma, soft tissue sarcoma and other malignancies. Per independent review committee assessment, pooled data from an expanded cohort demonstrated an ORR of 75%, with a complete response rate of 22% and partial response rate of 53%. The antitumour responses were durable across patients, with the median duration of response not reached and a wide range of individual response durations observed (176). In addition, Kummar et al. demonstrated that Larotrectinib can significantly improve the health-related quality of life compared to traditional chemotherapy or immune checkpoint inhibitors in *NTRK* fusion-positive tumors (177). Predominant TRAEs of this drug (incidence rate ≥ 20%) include elevated ALT, elevated AST and dizziness; other TRAEs such as fatigue, nausea, vomiting, constipation, diarrhea and cough were also observed, yet their incidences were all less than 20%. Most of these adverse reactions were mild, and few led to dose interruption or permanent treatment discontinuation (175).

#### 7.1.2 Entrectinib

Entrectinib is a new type of oral small molecule inhibitor that can specifically target three kinases: TRK, ROS1 and ALK, indicated for locally advanced or metastatic solid tumors harboring *NTRK1/2/3*, *ROS1* and *ALK* gene fusion mutations. It exhibits excellent BBB penetration, making it the only clinically validated TRK inhibitor effective for primary and metastatic brain diseases. Its mechanism involves competing with ATP for binding to the kinase active site, inhibiting catalytic activity to suppress tumor cell proliferation with anti-tumor efficacy validated in mouse models, human tumor cell lines, and xenografts (178–180). Paz-Ares et al. systematically evaluated entrectinib in 54 TRK inhibitor-naive *NTRK* fusion-positive NSCLC patients (pooled data from ALKA-372-001, STARTRK-1, STARTRK-2 trials). The results showed that an mPFS of 11.2 months and an ORR of 57% (7% CR, 50% PR). An additional 10 *NTRK* gene fusion-positive NSCLC patients achieved an ORR of 70% (10% CR, 60% PR). In terms of safety, the TRAEs of Entrectinib in the treatment of NSCLC are mainly mild grade 1–2 reactions, including dyspepsia, dizziness and constipation (181). Severe TRAEs are rare, with no treatment-related death cases. Only a small proportion of patients require dose reduction or discontinuation due to adverse events (182).

### 7.2 Second-generation TRK inhibitors

#### 7.2.1 Repotrectinib

Repotrectinib is a selective inhibitor of proto-oncogene tyrosine-protein kinases ROS1 and the TRK family (TRKA, TRKB, TRKC). ROS1 Fusion proteins drive tumorigenesis by overactivating cellular signaling, proliferation and division pathways, while oncogenic mutations promote unregulated cell division and downstream signaling dysregulation. Repotrectinib binds to specific sites on tyrosine kinase receptors, blocking phosphorylation to inhibit these abnormal pathways (183). The multicenter TRIDENT-1 trial enrolled *ROS1* fusion-positive NSCLC patients across four cohorts, all receiving Repotrectinib 160 mg once daily for 14 days, then 160 mg twice daily thereafter. Efficacy outcomes were as follows. In the first-line cohort of ROS1-TKI-naïve patients (n = 71), the ORR was 79%, with a mDoR of 34.1 months and a mPFS of 35.7 months. The intracranial response rate reached 89% among patients with measurable brain metastases. For patients treated with one prior ROS1-TKI without chemotherapy (n = 56), the ORR was 38%, mDoR 14.8 months, median PFS 9.0 months, and intracranial response rate 38%. The ORR was 42% in the cohort receiving one prior TKI plus chemotherapy (n = 26) and 28% in patients exposed to two prior TKIs. Notably, the ORR hit 59% in the subgroup with G2032R resistance mutation (n = 17). Among patients without baseline brain metastases, the 12-month rate of no new intracranial lesions was 91% (first-line) and 82% (prior TKI exposure). Among the 426 patients in the Phase II safety population, the most common adverse events included dizziness (58%), dysgeusia (50%) and paresthesia (30%), most of which were grade 1–2. The overall incidence of grade ≥ 3 adverse events was 29%, with anemia and elevated CPK each accounting for 4%. Dose reduction occurred in 38% of patients, treatment interruption in 50%, and permanent discontinuation in only 3%. Interstitial lung disease occurred in 3%, with no treatment-related deaths. Additionally, more than 60% of patients maintained or experienced improvements in quality of life scores during treatment (184).

#### 7.2.2 Zurletrectinib

Zurletrectinib is a novel, highly potent and highly selective next-generation Type I TRK inhibitor. A Phase I/II basket trial (NCT05745623) being conducted in China assesses its efficacy against advanced solid tumors and primary central nervous system tumors, and the full results are anticipated to be released in 2026. Preliminary data from 6 patients with *NTRK* fusion-positive tumors showed an ORR of 66.7% and a DCR of 100%. Notably, one patient with measurable brain metastases had the intracranial target lesion reduced from 10 mm to 3 mm. The drug demonstrates favorable tolerability, and further research will focus on its frontline application and acquired resistance mechanisms (171).

### 7.3 The mechanism of resistance to small molecule inhibitors targeting TRK

Resistance to TRK-targeted therapies is mainly categorized into two major mechanisms. On-target kinase mutations account for approximately 55% of resistance to first-generation TRK inhibitors, predominantly involving solvent-front G595R mutations (35% - 40%), gatekeeper mutations such as F589L/F617L (10% - 15%), and xDFG mutations including G667C/G667S (8%–12%). Bypass pathway activation driven by *KRAS/BRAF* mutations and *MET* amplification contributes to 35%–45% of cases. The remaining around 5% of resistance has no identifiable cause. For NSCLC, the ORR of first-generation Larotrectinib is 75% and that of Entrectinib is 70%. Both agents eventually fail due to the aforementioned kinase mutations and bypass activation. Second-generation Repotrectinib achieves an ORR of 40%–50% against G595R and gatekeeper mutations. However, all second-generation TRK inhibitors are intrinsically inactive against G667C. Patients treated with second-generation agents are also prone to developing secondary G667C mutations or new bypass abnormalities, leading to re-emergent resistance. Cabozantinib achieves an ORR of 25% - 35% for tumors harboring the G667C mutation. For bypass-mediated resistance, single-agent TRK inhibitors produce a response rate below 10%, while combination therapy of TRK inhibitors with MEK and other pathway inhibitors can increase the efficacy to approximately 30% (164).

## 8 Small molecule targeted drugs targeting RET

The RET proto-oncogene encodes a transmembrane tyrosine kinase receptor and is located on human chromosome 10 (185, 186). Upon ligand stimulation or during tumorigenesis, sustained RET activation induces autophosphorylation of its encoded tyrosine kinase receptor and initiates downstream signal transduction. This activates multiple cell survival pathways such as RAS/RAF, JAK-STAT, ERK1/2, and PI3K, which are involved in tumor initiation and progression (151). *RET* genetic alterations fall primarily into point mutations and gene fusions, with *RET* fusion being the main variant form in NSCLC, accounting for approximately 1% - 4% of NSCLC cases in the Chinese population (187). To date, over 40 *RET* fusion partner genes, such as Kinesin Family Member 5BNCBI (*KIF5B*), Coiled−Coil Domain Containing 6 (*CCDC6*), and Nuclear Receptor Coactivator 4 (*NCOA4*), have been identified. Among them, *KIF5B-RET* fusion is the most common type of primary *RET* fusion, while *CCDC6-RET* fusion represents the most prevalent acquired *RET* fusions secondary to EGFR/ALK TKIs resistance (188, 189). Patients with *RET* fusion-positive NSCLC exhibit typical clinical characteristics: relatively young age (< 60 years), never or light smoking history, adenocarcinoma as the main pathological type, and a high incidence of brain metastasis. These clinical features provide important guidance for the early identification, diagnosis, and treatment of the disease (190, 191). The summary of small molecule inhibitors targeting RET is shown in **Table 7**.

**Table 7:** Summary of small molecule inhibitors targeting RET.

Drug	Target	Adaptation disease	Adverse reaction (Grade ≥ 3)
Pralsetinib	RET	Indicated for adult metastatic RET-fusion positive NSCLC and radioactive iodine-refractory advanced metastatic thyroid carcinoma in patients aged ≥ 12 years (192).	Severe interstitial pneumonia, Grade ≥ 3 hypertension, serious hemorrhage, tumor lysis syndrome, accompanied by elevated liver enzymes plus decreased blood cell counts, serum calcium and phosphorus (192).
Selpercatinib	RET	Indicated for RET-fusion positive advanced NSCLC, progressive RET-mutant medullary thyroid carcinoma, and radioactive iodine-refractory advanced RET-fusion positive thyroid carcinoma (193).	Elevated liver enzymes, ILD, severe hypertension, QT prolongation, severe hemorrhage, serious hypersensitivity, tumor lysis syndrome, severe myelosuppression and gastrointestinal injury; slipped capital femoral epiphysis and severe hypothyroidism may occur in pediatric patients (193).

In the early stage of RET-targeted therapy, multikinase inhibitors (MKIs) such as Cabozantinib, Vandetanib, and Lenvatinib (trade name: Lenvima; hereafter referred to as Lenvatinib) were used for the treatment of *RET* fusion-positive NSCLC due to their moderate RET inhibitory activity. However, these drugs lack RET specificity, are prone to off-target effects, and are associated with significant toxicities and a low ORR, leading to their gradual withdrawal from first-line clinical use (194–196).

Up to now, the NMPA of China has approved two highly selective RET inhibitors, Pralsetinib (trade name: Pujihua; hereafter referred to as Pralsetinib) and Selpercatinib (trade name: Ruituo; hereafter referred to as Selpercatinib), for the treatment of patients with *RET* fusion-positive locally advanced or metastatic NSCLC, and relevant clinical studies have confirmed their definite efficacy (197). The latest data from the ARROW study showed that Pralsetinib achieved an ORR of 72% and 59%, and an mPFS of 13.0 months and 16.5 months in the first-line treatment and platinum-containing chemotherapy-refractory cohorts of *RET* fusion-positive NSCLC patients, respectively. For intracranial metastatic lesions, the intracranial ORR was as high as 70%, with an intracranial DoR of 10.5 months (198). The latest results of the LIBRETTO-001 study indicated that Selpercatinib yielded an ORR of 84% and 61%, and an mPFS of 22.0 months and 24.9 months, in first-line and platinum-containing chemotherapy-refractory patients, respectively. Its intracranial ORR reached 85% with an intracranial DoR of 9.4 months, demonstrating superior systemic and intracranial therapeutic efficacy (199). In terms of safety, both Selpercatinib and Pralsetinib are associated with a relatively high incidence of grade ≥ 3 hypertension (11% - 14%). Adequate blood pressure optimization is required before clinical treatment, with regular monitoring during therapy. Antihypertensive drugs should be initiated or adjusted in a timely manner when necessary to ensure treatment safety. In addition, Selpercatinib is associated with a relatively higher incidence of grade ≥ 3 hepatic toxicity, with ALT elevation and AST elevation occurring in 12% and 10% of patients, respectively (199). LIBRETTO-431 is a randomized Phase III trial evaluating the efficacy of first-line Selpercatinib for RET fusion-positive advanced non-squamous NSCLC. A total of 261 patients with advanced or metastatic *RET* fusion-positive non-squamous NSCLC confirmed by NGS or polymerase chain reaction (PCR) were enrolled. The experimental group received oral Selpercatinib 160 mg twice daily, while the control group was administered platinum plus Pemetrexed with or without Pembrolizumab. The initial randomization ratio of 1:1 was amended to 2:1 and finally adjusted to 1.6:1. Patients in the control group were permitted to cross over to Selpercatinib upon disease progression. The primary endpoint was PFS in the ITT population (n = 212) assessed by BICR. The median PFS was 24.8 months (95% CI: 16.9 to not evaluable) in the Selpercatinib group, significantly longer than 11.2 months (95% CI: 8.8–16.8) in the chemo-immunotherapy control group (HR = 0.46, 95% CI: 0.31–0.70, *p* < 0.001). The investigator-assessed PFS HR was 0.53, while the PFS HR for the overall study population was 0.48. Selpercatinib yielded an ORR of 84%, superior to 65% for chemo-immunotherapy and 63% for chemotherapy alone. Among patients with brain metastases, the intracranial ORR and intracranial complete response rate were 82% and 35% with Selpercatinib, versus 58% and 17% in the control group, with an intracranial progression HR of 0.28. Only 7% of patients in the targeted therapy group experienced first progression in the central nervous system, with a 12-month cumulative incidence of intracranial progression was 6%, compared with 18% and 20% in the control group, respectively (200).

In terms of safety, elevated liver function parameters were the most prominent adverse events. The incidences of grade ≥ 3 ALT elevation,AST elevation and bilirubin elevation were 22%, 13% and 1%, respectively. Most adverse events were manageable with dose reduction, and only 2% of patients discontinued treatment due to hepatotoxicity (200). In contrast, grade ≥ 3 neutropenia is the most common TRAE of Pralsetinib (incidence: 18%), and these TRAEs usually gradually alleviate after the 4th week of treatment (201). Therefore, regular hematological tests should be conducted during the treatment with these two drugs, especially in the early phase. When significant neutropenia occurs, granulocyte colony-stimulating factor (G-CSF) should be administered promptly or the drug dosage adjusted to mitigate TRAE risk.

To explore the resistance mechanisms of RET inhibitors, a study enrolled 109 patients with *RET-*mutated advanced tumors (94 cases of lung cancer and 15 cases of thyroid cancer) and analyzed 143 post-resistance specimens from patients treated with Selpercatinib or Pralsetinib. The results demonstrated that the overall resistance mechanisms of the two selective RET inhibitors were similar, with data not stratified by individual agent. The overall median PFS of first-line treatment was 13.9 months, and the median time to treatment discontinuation (TTD) was 17.3 months. The resistance mechanisms of RET can be mainly classified into three categories. First, secondary on-target *RET* mutations were identified in 14% of all cases, including 12.4% in lung cancer and 22.7% in thyroid cancer. G810 and V804M were the predominant mutations, which altered the spatial conformation of the kinase domain and impeded drug-target binding. *RET* amplification was detected in only 2.1% of specimens. Second, bypass pathway activation represented the leading cause of resistance. In paired samples, isolated bypass alterations accounted for 60.6%, while combined on-target mutations plus bypass alterations accounted for 10.1%. *MET* amplification was the most common bypass abnormality, with its incidence rising from 2% at baseline to 17.6% after resistance (overall bypass alterations: 18.2%; *MET* amplification: 15.2%). Patients with *MET* amplification had a median TTD of merely 8.4 months, significantly shorter than 19.0 months in those without MET abnormalities. Additionally, aberrations in *TP53, APC, KRAS, CDKN2A/B, EGFR, ALK* and *BRAF* drove resistance by bypassing the RET signaling pathway. Among all bypass alterations, 31.7% could be targeted by approved agents and 16.7% by investigational drugs. Third, non-genetic resistance was observed. No driver gene mutations were identified in 27.3% of samples. Histological transformation from lung adenocarcinoma to small cell lung cancer occurred in 1.3% of cases, whereby tumor cells escaped drug inhibition through phenotypic changes (202).

## 9 Small molecule targeted drugs targeting MET

The *MET* oncogene, located on chromosome 7q21 - q31, encodes a transmembrane receptor of the receptor tyrosine kinase (RTK) family. Activated by hepatocyte growth factor (HGF), MET regulates cell growth and development by modulating cell mitosis, migration and invasion. Pathological activation of MET can promote tumor cell proliferation, invasive growth and angiogenesis, thereby driving the progression of various malignant tumors (203). As a key driver gene of NSCLC, *MET* aberrations manifest in multiple forms, mainly including *MET* exon 14 skipping mutation (*MET* ex14), *MET* gene amplification, MET protein overexpression and *MET* fusion. Among them, the *MET* ex14 mutation is mostly a primary tumor driver variant with an incidence of approximately 1% - 3.6%, which is higher in the elderly, female, never-smoking populations, as well as in pulmonary sarcomatoid carcinoma and lung adenocarcinoma (204, 205). For *MET* aberrations in NSCLC, targeted therapy with selective small-molecule MET-TKIs or multi-target kinase inhibitors is currently available in clinical practice (151). Small molecule inhibitors targeting MET are shown in **Table 8**.

**Table 8:** Summary of small molecule inhibitors targeting MET.

Drug	Target	Adaptation disease	Adverse reaction (Grade ≥ 3)
Capmatinib	MET	Indicated for adult metastatic NSCLC harboring MET ex14 (206).	Dyspnea, pneumonia, edema, fatigue, elevated liver and pancreatic enzymes, hematologic and electrolyte abnormalities; permanent drug discontinuation is required for severe interstitial lung disease (206).
Tepotinib	MET	For adult metastatic NSCLC with MET ex14 (207).	May cause edema, pleural effusion, pneumonia, myalgia and dyspnea, accompanied by decreased hematological parameters, serum sodium and albumin as well as elevated hepatic and pancreatic enzymes; permanent discontinuation is needed for severe interstitial lung disease, liver injury or pancreatitis (207).
Savolitinib	MET	For locally advanced/metastatic NSCLC with MET ex14 (208).	AST elevation, ALT elevation, peripheral edema, hypokalemia, hypoalbuminemia, fever, anemia, and elevated serum creatinine (208).
Glesatinib	MET	Indicated for advanced NSCLC with MET exon14 skipping mutation after failure of chemotherapy or immunotherapy, as well as patients with secondary resistance to type I MET inhibitors caused by A-loop site mutations; not applicable for L1195V and F1200L resistant mutations (70).	/

### 9.1 Capmatinib

Capmatinib (trade name: Tuoruida; hereafter referred to as Capmatinib) is an oral, highly potent and highly selective MET inhibitor. Studies have confirmed that direct *MET* gene aberrations or HGF-mediated MET activation may induce tumor cell resistance to various kinase inhibitors (209). A Phase I clinical trial conducted in Japanese solid tumor patients (without subject selection based on MET status) showed that Capmatinib was well-tolerated and had definite clinical efficacy (210). The recently published multi-cohort Phase II GEOMETRY mono-1 study aimed to evaluate the efficacy of Capmatinib in the treatment of treatment-naive and pretreated advanced NSCLC patients with *MET* ex14 mutation. The results showed that the treatment-naive cohort achieved an ORR of 67.9%, a median DoR of 11.1 months and an mPFS of 9.6 months (211). Its TRAEs are mainly peripheral edema, nausea, fatigue, etc., and it also has potential risks of specific toxicities such as ILD/pulmonary inflammation and hepatic toxicity, as well as phototoxicity. The overall safety profile is acceptable in the context of the treatment of this disease (212).

### 9.2 Tepotinib

Tepotinib (trade name: Tepmetko; hereafter referred to as Tepotinib) is the first oral MET-targeted TKI, demonstrating significant efficacy in advanced NSCLC and liver cancer (213). As a selective MET inhibitor, Tepotinib suppresses melanoma cell proliferation, migration, invasion and epithelial-mesenchymal transition while facilitating cell apoptosis, and exerts anti-tumor activity via inhibiting the activation of MET and downstream PI3K/AKT signaling pathways (214). The VISION study (NCT02864992) specifically evaluated the long-term efficacy and safety of Tepotinib in NSCLC patients with *MET* ex14 skipping mutation. The results showed an overall ORR of 51.4% (95% CI: 45.8% - 57.1%) and a median DoR of 18.0 months (95% CI: 12.4 - 46.4 months) in all patients, with an incidence of grade 3 and above TRAEs of 34.8% (215). Common TRAEs of Tepotinib include edema, fatigue, nausea, diarrhea, musculoskeletal pain and dyspnea; the agent may also induce ILD, hepatic toxicity, and embryotoxicity, requiring close clinical monitoring (216).

### 9.3 Savolitinib

Savolitinib (trade name: Worisha; hereafter referred to as Savolitinib) is a highly potent, highly selective type Ib small-molecule MET tyrosine kinase inhibitor. It blocks the HGF/MET signaling cascade and its downstream pathways including PI3K/AKT, MAPK and NF-κB, thereby suppressing tumor cell proliferation, angiogenesis and malignant progression, and restraining sustained oncogenic signal transduction driven by *MET* aberrations (217). The results of the Phase III clinical trial (NCT04923945) disclosed in 2023 showed that in Cohort 2 of the study, which included treatment-naive locally advanced or metastatic NSCLC patients with *MET* ex14 skipping mutation, the ORR was 59.5% (95% CI: 48.3% - 70.1%) and the mPFS was 12.6 months (95% CI: 8.5 months-not reached), with 66.7% of patients experiencing grade 3 and above TRAEs (218). Savolitinib is associated with a relatively large number of TRAEs, the most common being nausea and vomiting, peripheral edema, hypoproteinemia, leukopenia, and abnormal liver function. However, most of these TRAEs are grade 1–2 with good clinical tolerability (219).

### 9.4 Glesatinib

Glesatinib is a TKI targeting both MET and AXL aberrations. *In vivo* experiments and clinical studies have confirmed its definite therapeutic activity in NSCLC patients positive for MET ex14 mutation, with efficacy also observed in patients with recurrence after Crizotinib treatment (70). A study of 68 patients with advanced NSCLC identified 28 and 8 cases of MET ex14 skipping mutation via tumor tissue and ctDNA testing, respectively, as well as 20 and 12 cases of MET gene amplification by tumor tissue and ctDNA testing, respectively. The overall ORR of all patients was 11.8%, with an mPFS of 4.0 months and a mOS of 7.0 months. Among MET-mutated patients, the response rates of those positive by tumor tissue and ctDNA testing were 10.7% and 25.0%, respectively; among MET-amplified patients, only those positive by tumor tissue testing had a response, with a response rate of 15.0%. Key drug-related TRAEs include diarrhea, nausea, elevated transaminases, and fatigue (220).

### 9.5 The mechanism of resistance to small molecule inhibitors targeting MET

Resistance to MET-TKIs (Capmatinib and Tepotinib) in MET-targeted therapy primarily falls into two categories. The first is MET-dependent resistance, accounting for approximately 35% of cases, which arises predominantly from secondary mutations in the MET kinase domain. As type Ib MET inhibitors, Capmatinib and Tepotinib mainly induce resistance mutations at the D1228 and Y1230 sites. Clinically, this type of resistance can be overcome by switching to MET-TKIs or macromolecular agent drugs such as Amivantamab. The second category is MET-independent resistance, which mainly includes bypass activation mediated by abnormalities in the *EGFR*, *HER2* and *KRAS* genes, sustained activation of the downstream PI3K signaling pathway (a common driver of primary resistance), and rare histological transformation of tumors into small cell lung cancer or squamous cell carcinoma (221).

A *post-hoc* ctDNA analysis of the phase II clinical trial (NCT02897479) of Savolitinib for non-small cell lung cancer with *MET* ex14 identified two major resistance mechanisms of Savolitinib. Conducted in 21 patients with disease progression after treatment, the analysis detected secondary on-target *MET* mutations (D1228H/N, Y1230C/H/S) in 14.3% of patients, while bypass pathway activation driven by aberrations in driver genes, including *KRAS*, *NRAS*, *BRAF*, *PIK3CA*, *FGF19*, and *TP53*, accounted for 42.9%. Among 66 patients with available baseline ctDNA samples, the *MET* ex14 mutation was detectable at baseline in 70% of cases (222).

As a type II MET inhibitor, Glecirasib is minimally affected by activation loop resistance mutations such as Y1230C/H/S that commonly emerge against type I MET inhibitors. Only certain compound mutations reduce its efficacy by approximately 4-fold. Primary resistance drivers for this agent include L1195V, F1200L/I, D1228N mutations and *MET* amplification. Among them, L1195V and F1200L/I are shared resistance sites for both type I and type II MET inhibitors; the D1228N mutation directly confers complete resistance to glecirasib, while *MET* amplification further exacerbates drug resistance. Animal model studies verify that Glecirasib induces tumor resistance much later than type I MET inhibitors. Most clinically resistant cases present with complex resistance characterized by the co-occurrence of the aforementioned multiple mutations and *MET* amplification (70).

## 10 Small molecule inhibitors based on new targets

In recent years, advances in research on targeted therapies for lung cancer have led to the continuous development of new agents. Small-molecule inhibitors targeting new targets are summarized in **Table 9**.

**Table 9:** Summary of small molecule inhibitors based on new targets.

Drug name	Target	Adaptation disease	Adverse reaction (Grade ≥ 3)
Daraxonrasib	RAS	Indicated for pretreated metastatic pancreatic cancer and advanced NSCLC with RAS mutations; preclinical data suggests potential utility in solid tumors such as colorectal carcinoma and RAS-mutated malignancies resistant to KRAS-G12C inhibitors (223).	/
Sevabertinib	HER2	Indicated for adults with locally advanced or metastatic non-squamous NSCLC harboring activating mutations in the HER2 tyrosine kinase domain confirmed by FDA-approved companion diagnostic testing who have progressed following prior systemic therapy (224).	Hypokalemia, elevated lipase, lymphopenia, hyponatremia, increased amylase, elevated AST and ALT (224).
Zongertinib	HER2	For the treatment of unresectable or metastatic non-squamous NSCLC in adult patients (225).	Laboratory abnormalities include lymphopenia, elevated ALT, AST and γ-Glutamyl Transferase(GGT), hypokalemia, and neutropenia. Clinically significant serious adverse reactions consist of pulmonary embolism, dyspnea, pneumonia, pleural effusion and pericardial effusion (225).
AZD0424	Src kinase	Chronic myeloid leukemia (CML), acute lymphoblastic leukemia (ALL), and multiple solid tumors including breast cancer and lung cancer (226).	/

### 10.1 Newly developed drugs targeting RAS

For example, Rayvotech Pharmaceutical has successfully discovered and developed the clinical candidate drug Daraxonrasib (Also known as RMC-6236; hereafter referred to simply as Daraxonrasib) using its continuously optimized R&D system (227, 228). Daraxonrasib is an oral, multi-selective inhibitor that directly acts on active RAS (ON). It can block the binding of RAS (ON) to downstream effector proteins, thereby inhibiting the RAS signaling pathway. This agent targets oncogenic RAS mutations including G12X, G13X and Q61X, and is indicated for multiple malignancies driven by these mutations, such as pancreatic cancer, NSCLC and colorectal cancer. Currently, two Phase III clinical trials of Daraxonrasib are underway for the treatment of pretreated metastatic PDAC (NCT06625320) and RAS-mutated NSCLC (NCT06881784), respectively (229, 230). Recent studies have also reported non-covalent reversible small-molecule multi-target RAS inhibitors that can regulate the binding affinity to active and inactive RAS (231, 232). The development of next-generation KRAS inhibitors that can target various oncogenic KRAS mutations without affecting wild-type HRAS and NRAS is expected to yield a new generation of precision KRAS inhibitors, achieving a balance between broad-spectrum anti-tumor activity and good safety.

### 10.2 Newly developed drugs targeting HER2

Sevabertinib is a reversible HER2 inhibitor that blocks the ATP-binding site and inhibits downstream signaling pathways to exert anti-tumor effects. After molecular optimization, it has low off-target toxicity. Preclinical data have confirmed its robust efficacy against NSCLC harboring HER2 exon 20 mutations. A total of 209 patients with HER2-mutated NSCLC were enrolled in a clinical trial conducted from October 25, 2021, to December 24, 2024, and all received Sevabertinib at a dose of 20 mg twice daily. Patients were divided into three study cohorts based on prior treatment history: Cohort D (n = 81) with prior systemic therapy but no prior HER2-targeted therapy; Cohort E (n = 55) previously treated with HER2-targeted ADCs; and Cohort F (n = 73) with locally advanced or metastatic disease who had not received first-line systemic therapy. As of June 27, 2025, all 209 patients had completed their assigned treatment. The median follow-up duration was 13.8 months, 11.7 months, and 9.9 months for Cohorts D, E, and F, respectively. Efficacy analyses showed the following results: Cohort D achieved an ORR of 64% (95% CI: 53 - 75), a median DoR of 9.2 months (95% CI: 6.3 - 13.5) and an mPFS of 8.3 months (95% CI: 6.9 - 12.3); Cohort E had an ORR of 38% (95% CI: 25 - 52), a median DoR of 8.5 months and an mPFS of 5.5 months; Cohort F yielded an ORR of 71% (95% CI: 59-81) and a median DoR of 11.0 months, with immature PFS data. Safety assessments revealed that grade ≥ 3 TRAEs occurred in 31% of patients. Diarrhea was the most common TRAE, with an overall incidence of 84% - 91%, grade ≥ 3 diarrhea, was observed in 5%–23% of cases. Only 3% of patients discontinued treatment due to TRAEs (233).

Zongertinib (Also known as BI 1810631; hereafter referred to as Zongertinib) is a highly selective irreversible covalent HER2 tyrosine kinase inhibitor developed by Boehringer Ingelheim. It potently inhibits HER2 and demonstrates high selectivity against wild-type EGFR, which reduces adverse events such as rash and diarrhea. The recommended standard dose is 120 mg administered orally once daily. This compound is active against various *HER2* mutations, *HER2* amplification, and *NRG1* fusions, and retains efficacy in tumors resistant to trastuzumab deruxtecan (T-DXd). Findings from the Beamion LUNG-1 trial are as follows. Among 75 heavily pretreated patients with HER2 kinase-mutant NSCLC who had no prior exposure to HER2 ADCs, the ORR was 71% and DCR 96%, with a mDoR of 14.1 months and median PFS of 12.4 months. In 31 patients with T-DXd resistance, the ORR reached 48% and DCR 97%. For 20 pretreated patients harboring non-kinase domain *HER2* mutations, the ORR was 30% and DCR 65%. In the cohort of 74 treatment-naive patients, the ORR was 76%, with a median DoR of 15.2 months and a median PFS of 14.4 months. The incidence of grade ≥ 3 TRAEs was low across all cohorts, and no treatment-related interstitial lung disease was reported throughout the study. Zongertinib has also demonstrated promising antitumor activity in clinical trials for other solid tumors, such as *HER2*-mutant breast cancer and *NRG1*-fusion-positive cholangiocarcinoma. Currently, this drug has been approved domestically and internationally for first-line and subsequent-line treatment of *HER2*-mutant NSCLC (234).

### 10.3 Newly developed drugs targeting Src kinase

Src kinase is a potential therapeutic target in NSCLC, as it is abnormally expressed and activated in this malignancy. It regulates core biological processes such as tumor cell proliferation, survival, and migration. In addition, upregulated Src expression and overactivated Src signaling are closely linked to resistance to EGFR-targeted therapies in NSCLC. AZD0424 is a highly selective oral dual inhibitor of Src and Abelson. Its IC_50_ against Src/ABL1 kinases is approximately 4 nM. Compared with vascular endothelial growth factor receptor 2 (VEGFR-2) and C−terminal Src kinase (CSK), its selectivity ratios exceed 933-fold and 448-fold, respectively, outperforming other similar agents such as Dasatinib and Bosutinib. Preclinical studies adopted a rat xenograft model established using the human Calu−6 lung cancer cell line (classified within NSCLC). AZD0424 exhibited moderate tumor growth inhibition in this model. At a daily oral dose of 20 mg or higher, the compound exerted robust targeted inhibition on Src. From week 2 to week 7 of administration, it induced sustained reductions in bone turnover markers, including urinary NTX and serum CTX, with superior inhibitory effects relative to the Src inhibitor saracatinib. The first-in-human Phase I clinical trial of AZD0424 enrolled 41 patients with advanced solid tumors, among whom 3 had lung cancer (7% of the total). No further stratification of lung cancer subtypes was performed in the study. AZD0424 displayed favorable pharmacokinetic properties: rapid oral absorption with Tmax < 2 h (dose-independent), a terminal half-life of 8.4 ± 2.8 h, dose-proportional increases in maximum plasma concentration (Cmax) and area under the curve (AUC), and high bioavailability. At equivalent doses, its plasma concentrations were significantly higher than those of Saracatinib. In terms of safety, TRAEs in patients with lung cancer were consistent with those observed in other solid tumor populations, with no unique toxicities identified. Overall treatment adherence was satisfactory: 68% of patients received at least 75% of the planned dose during the first cycle. No lung cancer-specific adverse events or unusual dose delays or adjustments were documented (235).

## 11 Conclusion

Small-molecule targeted therapy has emerged as a core modality for individualized treatment of advanced NSCLC. Targeted inhibitors against driver genes, including EGFR, ALK, KRAS, ROS1, BRAF, and MET, have undergone successive generational advancements in clinical practice. Based on our core research hypothesis, this review systematically categorizes clinically approved agents and summarizes preclinical and early-stage investigational drugs for novel targets, aiming to address the gaps in existing literature (**Table 10**). Most prior reviews predominantly focus on well-established marketed TKIs for mainstream targets. By contrast, this work consolidates research progress on understudied emerging targets (e.g., RAS, HER2, Src) as well as their corresponding small-molecule inhibitors (e.g.,Daraxorasib, Sevabertinib, Zongertinib), which constitutes a major innovative aspect of this study.

**Table 10:** Summary of small molecule targeted drugs that have been approved for NSCLC treatment.

Target	Drug	Main clinical data
EGFR	Gefitinib	mPFS: 12–14 months (26)
	Erlotinib	ORR: 35.29% mPFS: 12.6 months (31) mOS: 11.6 months (36)
	Icotinib	ORR: 27.6% mPFS: 4.6 months (38)
	Afatinib	ORR: 11.43% (43)
	Dacomitinib	mPFS: 18.4 months mOS: 34.1 months (44)
	Osimertinib	mPFS: 38.6 months (50)
ALK	Crizotinib	Phase I PROFILE−1001: post-line ORR 60.8%, mPFS 9.7 months Phase II PROFILE−1005: post-line ORR 53%, mPFS 8.5 months Phase III PROFILE−1007 (2L vs chemo): Crizotinib ORR 65%, mPFS 7.7 months (73)
	Brigatinib	Asian: mPFS 24.0 months, ORR 80%, DoR 22.2 months; intracranial ORR 62% for baseline brain metastasis Non-Asian: mPFS 24.7 months, ORR 71%, DoR 41.4 months; intracranial ORR 69% for baseline brain metastasis (76)
	Ceritinib	mPFS: 16.6 months (79–81)
	Ensartinib	mPFS: 25.8 months ORR: 75% (84)
	Alectinib	mPFS:25.7 months (87, 88)
	Lorlatinib	PFS:64% (91)
KRAS	Sotorasib	ORR: 37.1%, mPFS: 6.8 months mOS: 12.5 months (236).
	Adagrasib	mPFS:5.49 months (111)
ROS1	Crizotinib	ORR: 72% mPFS: 19.3 months mOS: 51.4 months (118)
	Entrectinib	ORR: 77% mPFS: 19.0 months (130)
	Brigatinib	mPFS:24 months ORR:74% (75, 76)
	Repotrectinib	ORR: 79% mPFS: 34.1 months mOS: 35.7 months (133)
	Taletrectinib	ORR: 66.7% mPFS: 29.1 months (136)
	Lorlatinib	ORR: 61% mPFS: 21.0 months (138)
BRAF	Dabrafenib	Dabrafenib Combined with Trametinib First-line: ORR 64%, mPFS 10.8 months, mOS 17.3 months Subsequent-line: ORR 68%, mPFS 10.2 months, mOS 18.2 months (160)
	Encorafenib	Encorafenib Combined with Binimetinib Pretreated pts: ORR:46% mPFS: 9.3 months (162).
TRK	Larotrectinib	ORR: 79% mPFS: 28.3 months mOS: 44.4 months (175).
	Entrectinib	mPFS:11.2 months ORR:57% (181)
	Repotrectinib	Treatment-naive 1L ROS1 pts: ORR 79%, mPFS 35.7 months Prior 1 ROS1-TKI without chemo: ORR 38%, mPFS 9.0 months Prior 1 TKI + chemo: ORR 42%; prior 2 TKIs: ORR 28% G2032R-resistant subgroup: ORR 59% (184)
RET	Pralsetinib	ORR: 72% mPFS: 13.0 months (198)
	Selpercatinib	ORR: 84% mPFS: 22.0 months (199)
MET	Capmatinib	ORR: 67.9% mPFS: 9.6 months (211)
	Tepotinib	ORR: 51.4% (215)
	Savolitinib	ORR: 59.5% mPFS: 12.6 months (218)

Previous studies have repeatedly summarized the development and clinical profiles of the first- to third-generation classic TKIs, which serve as mainstream therapeutic targets. In contrast, this review supplements the latest clinical data on newly developed agents. By offering distinct collative insights, it enriches the existing literature system of NSCLC targeted therapy. This work provides numerous practical insights for clinical practitioners and basic pharmaceutical researchers, this review delivers multiple practical beneficial inspirations: Clinicians can refer to the summarized emerging targeted therapeutic regimens to formulate individualized follow-up treatment plans for patients with multi-line resistance to conventional TKIs; preclinical drug developers obtain clear candidate target trajectories centered on RAS/HER2/Src, and can draw upon combination therapy strategies outlined herein to design novel combination-based preclinical trials.

Currently, marketed small-molecule targeted agents still face significant challenges, including acquired heterogeneous resistance, inadequate blood-brain barrier penetration and suboptimal control of grade ≥ 3 adverse events. Additionally, many novel target agents remain confined to preclinical or early phase clinical development without official marketing authorization. From a future development perspective, three core directions are identified based on collated literature data: First, accelerate translational development of fourth-generation EGFR-TKI, next-generation ALK/ROS1 inhibitors, and novel KRAS non-covalent inhibitors to address unmet clinical demands of drug-resistant patients; Second, optimize the molecular structures of existing small-molecule compounds to enhance intracranial permeability, catering to the treatment requirements of NSCLC patients with brain metastasis; Third, advance original research on innovative drugs targeting rare driver mutations and explore multi-target synergistic combination strategies to delay the onset of drug resistance.

With continuous advancements in liquid biopsy and high-throughput genetic testing technologies, precise molecular typing of NSCLC will further facilitate the widespread adoption of individualized targeted therapy. Artificial intelligence (AI) has emerged as a pivotal transformative tool for advancing precision diagnosis and treatment of lung cancer. Leveraging eight major omics datasets, including genomics, transcriptomics, radiomics, and pathomics, AI technologies enable comprehensive and high-precision applications covering early lung cancer screening, individualized therapeutic strategy formulation, dynamic therapeutic efficacy monitoring, and novel drug development. Multimodal data integration has been recognized as the core developmental trend in the field, facilitating the full-process penetration of AI in the entire lung cancer diagnosis and treatment workflow. Despite these promising advances, the clinical translation of AI-based lung cancer diagnosis and treatment systems remains confronted with multiple bottlenecks, including non-standardized data acquisition and collation, limited interpretability of deep learning models, insufficient generalized verification of algorithm frameworks, as well as inherent data privacy risks and algorithmic bias issues. To address these limitations, targeted strategies have been proposed, namely the establishment of unified standardized data specifications, the adoption of explainable artificial intelligence (XAI) techniques such as SHAP and Grad-CAM, the implementation of multi-center external validation cohorts, and the optimization of full-cycle data security management. Furthermore, human–machine collaboration is highlighted as the optimal paradigm for future clinical AI deployment. In clinical practice, AI should function exclusively as a physician-assisted tool, and all AI algorithms and predictive models must undergo rigorous clinical validation and standardized compliance supervision prior to large-scale clinical popularization and application (237).

The collated content herein not only complements existing review literature but also provides referential data and developmental insights for subsequent basic research, new drug screening and clinical trial design for small-molecule targeted drugs in NSCLC.
